# Impact of an mHealth App on Digital Transformation: Randomized Clinical Trial on Strengthening Digital Skills in Older Women

**DOI:** 10.2196/76725

**Published:** 2026-06-30

**Authors:** Laura Thais Del Vecchio Sampaio, Carlos Roberto Bueno Júnior, Andressa Crystine da Silva Sobrinho, Grace Angélica de Oliveira Gomes

**Affiliations:** 1 Departamento de Gerontologia, Universidade Federal de São Carlos, Rodovia Washington Luís 310, São Carlos, 13565- 905, Brazil, +55 16 3306 666; 2 Faculdade de Medicina de Ribeirão Preto, Universidade de São Paulo, Ribeirão Preto, Brazil

**Keywords:** competencies, digital health, elderly people, gerontology, intervention, lifestyle habit, mHealth, mobile health, mobile phone

## Abstract

**Background:**

The rapid growth of digital technologies has transformed daily activities, health management, and social interaction. Older adults, however, continue to face challenges in adopting and using these tools due to limited previous exposure, age-related sensory or cognitive decline, and low digital confidence. In Brazil, internet access among adults aged  60 years or older has increased, yet digital exclusion persists, worsening health disparities. Mobile health (mHealth) apps offer a potential strategy to promote digital inclusion, strengthen digital competencies, and support healthy aging. Nonetheless, studies show that culturally adapted, multidisciplinary interventions for this group remain scarce and are rarely assessed through both quantitative and qualitative methods.

**Objective:**

This study aimed to evaluate the impact of a lifestyle mHealth app on improving digital skills, as well as to analyze the level of satisfaction and usability of the app.

**Methods:**

In this mixed methods study, a 14-week randomized clinical trial was conducted in Ribeirão Preto, São Paulo, Brazil. A total of 40 older adult women were randomized into an intervention group (n=21), who used the mobile app, and a control group (n=19). Digital competencies were measured before and after the intervention using a semistructured questionnaire based on the *Modelo de Competências Digitais para M-learning com foco em idosos* (MCDMSênior; Digital Competency Model for M-learning with a focus on older adults) framework, covering 6 domains—basic technology use, internet navigation, mobile app use, online research, digital communication, and usage of digital resources. Additionally, satisfaction with the educational content was evaluated using the suitability assessment of materials, and system usability was assessed using the System Usability Scale. Qualitative data were collected through semistructured, in-person interviews conducted immediately after the intervention with all intervention participants. Interviews explored perceptions of the app’s usability, satisfaction with its content, barriers, and facilitators to engagement, and perceived changes in digital skills. All interviews were audio-recorded, transcribed, and analyzed thematically by 2 independent researchers using an inductive coding approach.

**Results:**

Postintervention analyses revealed significant differences in specific digital competencies. The intervention group demonstrated a moderate improvement in internet navigation skills, while gains in basic technology use and digital communication were minimal. Conversely, the control group exhibited moderate improvement in basic technology skills and lower effects in online research and digital communication. Overall, satisfaction with the educational content was low, and usability was rated as average. Qualitative findings indicated that, although participants valued the clarity of navigation and cultural relevance, persistent age-related fears and insecurities in using digital technologies were reported. Participants highlighted the need for more personalized guidance, ongoing motivational support, and technical adjustments to improve usability and engagement.

**Conclusions:**

mHealth apps can effectively enhance certain digital competencies in older women, particularly internet navigation, but improvements in content suitability and usability are needed. Refinements in design and tailored support are essential to overcome age-related barriers and foster digital inclusion.

## Introduction

The COVID-19 pandemic acted as a “great accelerator,” dramatically speeding up digital transformation across sectors and compressing years of technological progress into months [1]. This rapid advancement has brought significant challenges and opportunities for the older adult population [2]. Increased social isolation, alongside growing demands on limited health services, cost containment pressures, and evidence-based recommendations supporting their adoption, has collectively driven the integration of health technologies [3-5].

Evidence shows that in Brazil, 58% of adults aged 50‐80 years reported using internet-based health and wellness services during 2022, compared with 37% in 2019 [6]. This increase indicates a greater acceptance of telehealth among older adults, highlighting the potential for expansion of these services [7]. Therefore, it is crucial to continue advancing digital integration efforts to ensure that older adults can benefit from these services [8].

Despite these advances, the older adult population still faces significant barriers in adopting digital technologies. Studies highlight previous experience, digital exclusion, and cognitive and motivational barriers [9,10]. Other critical obstacles are usability and user satisfaction, which affect digital adoption among seniors [11]. Therefore, it is essential to address these barriers with targeted interventions and support to overcome the digital divide and facilitate older adults’ engagement with digital skills [11-13].

Digital competencies are defined as skills, knowledge, and attitudes crucial to the effective use of digital technologies, from basic device operation to evaluating and communicating online content, benefiting the health and well-being of the older adult population [14,15]. Recent evidence demonstrates that higher levels of digital competence are consistently associated with increased adoption of telehealth services across different contexts and populations. Such competence not only facilitates access to online health resources but also enhances users’ confidence in navigating digital systems, thereby reducing the risk of exclusion and mistrust. Moreover, systematic reviews highlight that insufficient digital skills remain a primary barrier for older adults, reinforcing that targeted strategies to strengthen digital literacy are crucial to mitigate inequities in access and health outcomes [15-17].

Previous systematic reviews have also emphasized the importance of assessing digital competencies to understand barriers and facilitators of technology adoption among older adults [15]. In addition, recent evidence shows that greater digital competence is consistently associated with higher telehealth usage, reinforcing the importance of addressing disparities in digital literacy [16]. Thus, this research specifically investigates how a mobile app that promotes lifestyle habits can improve digital skills in older adults, in addition to analyzing usability and user satisfaction.

## Methods

### Overview

This fragment of the study was conducted as a mixed methods design, integrating quantitative and qualitative approaches in order to capture both statistical outcomes and participants’ subjective experiences.

### Type of Study

This project is a fragment of a larger 14-week randomized clinical trial aimed at developing, validating, and testing a multidisciplinary mobile app to promote lifestyle changes in older adults through simple, personalized guidelines tailored to their daily needs. The broader project encompassed a user-centered design process with iterative prototyping, usability testing, and active involvement of older adults in co-design sessions to ensure accessibility, intuitive navigation, and cultural appropriateness of the interface [18,19].

The app integrates multidisciplinary telecare resources, enabling interaction with medical, nursing, nutrition, psychology, and physical education professionals, and offers educational content, health monitoring tools, and motivational strategies such as personalized feedback and social engagement features. In addition to supporting the formation of healthy habits, the platform was designed to address common barriers in this population, including low digital literacy, fear of technology, and the need for ongoing guidance. This was achieved by incorporating progressive integration, adjustable font sizes, and simplified navigation flows [18,19].

To ensure data reliability, privacy, and anonymity, the system uses end-to-end encryption for data in transit, secure hashing of credentials, and encrypted storage in a HIPAA (Health Insurance Portability and Accountability Act)-compliant cloud database. The backend (Node.js/TypeScript) communicates with the frontend via a secure API (HTTPS/TLS 1.3), with all personal data anonymized or pseudonymized before analysis. Access is restricted through role-based permissions with audit logs, and the managed MongoDB Atlas cluster provides automated backups, redundancy, and disaster recovery [18].

### Location, Period, and Participants

The research was carried out in older adults living in the city of Ribeirão Preto, in the interior of the state of São Paulo, Brazil. It was carried out between July and December 2023. Participants were recruited through posters displayed in strategic locations, social media outreach, and press coverage. Interested participants called the research team, expressed their interest, and verified their eligibility.

The inclusion criteria were older adult individuals aged 60 years or older who owned a mobile device compatible with the app. The app was developed for both Android (Google) and iOS (Apple Inc) operating systems, and it was tested on all models owned by participants, functioning without technical restrictions. Internet connectivity (via Wi-Fi or mobile data) was required to access the educational content and interactive features of the app, ensuring full usability and synchronization of data. The exclusion criteria included limitations in carrying out the assessments included in the project, such as visual and auditory limitations and severe cognitive impairments, assessed by the Montreal Cognitive Assessment instrument [20].

Participants were randomly allocated to the intervention and control groups in a 1:1 ratio using a computer-generated randomization sequence (Microsoft Excel RAND function). Randomization was performed independently by a researcher who was not involved in recruitment or outcome assessment to ensure allocation concealment. The sequence was generated before the start of the study, and group assignments were revealed only after baseline assessments were completed. No stratification was used, given the homogeneity of the sample in terms of age range and socioeconomic characteristics. This randomized clinical study followed the guidelines of the CONSORT (Consolidated Standards of Reporting Trials) [21]. Initially, 70 volunteers were assessed for eligibility; 20 were files that did not meet the criteria or for other reasons. The 50 eligible participants were randomized into 2 groups (app: n=25; control: n=25). There were 10 losses to follow-up in the intervention group, resulting in 40 cases analyzed.

Outcome assessors responsible for conducting the pre- and postintervention evaluations were blinded to group allocation and study objectives. Participants were not blinded due to the nature of the intervention. Randomization and allocation were managed by an independent researcher not involved in the assessments.

The sample size of causes and effects, with a minimum sample size of 34 people, was calculated using the G*Power 3.1 software, using ANOVA for inter- and intragroup interaction with the following input parameters: effect size of 0.25, Type I error=0.05, type II error=0.80, number of groups=2, and number of measurements=2. A standard correlation between the measurements of 0.50 and a dropout rate of 20% were also used to account for possible sample losses. The use of an effect size of 0.25 was based on a similar study, which investigated the effects of using a minimally supervised app in community-dwelling older adults compared with an unsupervised model [22].

### Research Development

At the beginning of the intervention, a detailed meeting was held to introduce the app, explore its functionalities, and clarify operational doubts. During the first week, the researchers maintained daily contact with the intervention group, offering ongoing support to facilitate familiarization with the mobile app. The frequency of contact was adjusted to every other day in the second week and subsequently to biweekly intervals, aiming to reduce technical difficulties and assess independent adherence to the app interface.

This monitoring strategy was designed to overcome barriers to use due to digital limitations and to monitor genuine participant adherence. The effectiveness of the intervention was measured through questionnaires and direct observations, focusing on changes in users’ digital skills and satisfaction with the app’s usability and content.

### Instruments

To meet the objectives of this study, several data collection instruments were used. The questionnaires covered many aspects, including sociodemographic characteristics, digital skills, economic classification, material satisfaction, and usability assessment. An anamnesis script was prepared to accompany the questionnaires, aiming to obtain data on the participants’ sex, date of birth, and years of education.

The Brazilian Economic Classification Criteria were used to categorize Brazilian families into economic classes (A, B, C, D, and E). This system assesses socioeconomic status through a comprehensive questionnaire. It considers indicators such as ownership of durable goods, the head of household’s education level, and access to essential public services. This methodology provides a more accurate social stratification than income surveys alone, considering the high variability and overlap in income estimates. Reference values for monthly household income illustrate the characteristics of these strata (eg, class A: BRL $21,826.74; B1: BRL $10,361.48; B2: BRL $5755.23; C1: BRL $3276.76; C2: BRL $1965.87 [approximately BRL $5=US $1 as of 2023]), clarifying the distinctions defined by this criterion [23].

A semistructured questionnaire based on the MCDMSênior (*Modelo de Competências Digitais para M-learning com foco em idosos*; Digital Competency Model for M-learning with a focus on older adults) framework was applied to assess the impact on improving the digital skills of the participants. This model was originally developed to evaluate digital competencies in older adults in mobile learning and eHealth contexts, with specific emphasis on inclusion and age-related challenges [14]. It encompasses six domains, each of which was analyzed separately in this study: (1) basic knowledge of technology – such as turning on or off the device, using the keyboard, and installing apps; (2) internet navigation skills – including browsing websites and accessing online content; (3) use of mobile apps – focused on downloading, opening, and handling apps; (4) ability to conduct online research – related to searching for health or general information on the internet; (5) digital communication – using text, voice, or video messages; and (6) use of digital resources – involving storage, downloads, and use of media files. The choice of this model is justified by its conceptual breadth and its direct alignment with the digital skills mobilized during the use of the app, such as navigation, interaction, and consumption of educational content.

Although the MCDMSênior was not yet a formally validated instrument at the time of data collection, its theoretical adherence and practical relevance provided strong justification for its use in this study. Preliminary pilot testing also indicated that participants were able to understand and respond to the items appropriately, supporting its feasibility in this context. The responses were scored on a 3-point scale; 1 indicated that the participant could perform the skill without difficulty, 2 indicated they could perform the skill but with difficulty, and 3 indicated they were unable to perform the skill.

Participants’ satisfaction with the app’s educational content was measured using the suitability assessment of materials (SAM), originally developed in English and later translated and adapted to Portuguese [24]. The SAM was included to evaluate whether the educational content provided in the app was understandable, culturally appropriate, and motivating for older adults, thereby ensuring that usability findings could be interpreted in light of content adequacy. The SAM consists of a checklist with 30 items, divided into 6 categories: content, text comprehension, illustration, presentation, motivation, and cultural adaptation. The score is assigned as follows: 2 points for “excellent” responses, 1 point for “adequate,” and 0 points for “inadequate.” The results are interpreted according to the scale: 0%‐39% indicates unsuitable quality, 40%‐69% adequate, and 70%‐100% superior [25].

To complement this assessment, the content validation index (CVI) was calculated to quantify interrater agreement regarding the relevance and clarity of each SAM item. The CVI was introduced to provide an additional layer of rigor, ensuring that the judgments about the educational content were not only based on participant feedback but also on expert consensus. This content analysis was performed both with a focus group and with participants from the intervention group, ensuring a comprehensive and comparative assessment of item validity. The CVI classifies scores into 6 levels: unacceptable (≤0.50), poor (>0.50 and≤0.60), mediocre (>0.60 and≤0.70), average (>0.70 and≤0.80), good (>0.80 and≤0.90), and excellent (>0.90). This approach allowed the identification of areas for improvement in the educational material and ensured robust interpretation of the data obtained [26].

User experience with the app was assessed using the System Usability Scale (SUS), originally developed by John Brooke and adapted for the Brazilian context [27]. Composed of 10 items, the SUS investigates aspects such as ease of use, speed of learning, efficiency, and overall satisfaction with the system. The SUS was applied exclusively to the intervention group, as only these participants had direct experience with the app, and was used to complement the SAM results by providing a measure of usability in parallel to content adequacy.

The SUS score is categorized as follows: “worst imaginable” (up to 20.5), “poor” (21 to 38.5), “average” (39 to 52.5), “good” (53 to 73.5), “excellent” (74 to 85.5), and “best imaginable” (86 to 100) [28]. In line with international interpretations, a score of approximately 71.5 is generally considered “above average,” reflecting satisfactory usability. In parallel, the CVI was also used to assess the consistency of the SUS items, applying the same content validity classification as described for the SAM.

All participants in both groups completed the pre- and postassessment instruments, except for SAM and SUS, as the control group did not use the intervention app. Monitoring and contact with participants were carried out to assist in understanding the app, minimize the risk of bias due to lack of familiarity, and reduce potential dropouts, a justification that also applied to the qualitative interviews. The qualitative interviews were conducted after the end of the intervention period, while the questionnaires and interviews took place at the University of São Paulo in Ribeirão Preto, the institution where the research originated. Volunteer students participated in filling out the questionnaires.

### Ethical Considerations

The study followed the principles of the Declaration of Helsinki. The research was ethically approved by the Research Ethics Committee of the University of São Paulo in Ribeirão Preto (registration CAAE 58433922.0.0000.5659) and registered with the Brazilian Clinical Trials Registry (RBR-6wgkzs8). All participants signed the informed consent form, being informed about the research, its minimal risks, and their rights, including the option to withdraw without consequences. To ensure the privacy and confidentiality of participants, all data collected were anonymized and deidentified before analysis. Personal information was treated with the utmost confidentiality, and access to the raw data was restricted to authorized research staff only. There was no financial or other compensation for study participants. Participation was voluntary and based on an interest in contributing to the research. The manuscript does not contain images or supplementary materials that allow for the individual identification of participants. All figures and tables present aggregated data, ensuring the protection of the identity of those involved.

### Statistical Analysis

Data were processed and analyzed using IBM SPSS Statistics 25.0 and Microsoft Excel 2019. For a complete view of the data, measures of central tendency and dispersion, such as mean and SD, were calculated, as well as representations in percentage values. Initial similarity between groups was analyzed with Student *t* test for socioeconomic variables, years of education, and age.

An intention-to-treat (ITT) analysis was performed using up to 5-level multiple imputations to address missing data and preserve the ITT principle. In this approach, all participants were included according to their original randomization, regardless of treatment adherence or losses to follow-up. Multiple imputation was applied to create 5 complete datasets, thereby minimizing potential bias due to missing data. This procedure was adopted because ITT analyses provide a more conservative and realistic estimate of intervention effects in clinical trials [29,30].

To examine the effects of time (pre vs post), group (intervention vs control), and the interaction between them (time×group) on the outcome measures, generalized linear mixed models (GLMM) were used. GLMMs were chosen because they are well-suited for analyzing repeated measures data, account for intraindividual correlations, and are robust to unbalanced datasets and missing values [31,32].

Adjustment for potential confounders was conducted to increase model precision and control for variables known to influence both digital competence and technology adoption among older adults. Specifically, age, sex, education, and socioeconomic status were included as covariates, given their established associations with digital literacy and health technology use [9,12].

Model fit was assessed using the Akaike Information Criterion (AIC), a widely applied metric that balances model fit and parsimony, penalizing for unnecessary complexity [33,34]. Moreover, 4 covariance structures were tested, and the model with the lowest AIC was selected, which corresponded to the diagonal covariance matrix.

All analyses were conducted with 95% CIs and a 5% significance level. For multiple comparisons, Bonferroni post hoc tests were applied. To support clinical interpretation, Cohen *d* effect sizes were calculated and categorized as small (Cohen *d*=0.2), medium (Cohen *d*=0.5), large (Cohen *d*=0.8), or very large (Cohen *d*>1.0).

## Results

### Quantitative Results

**Figure 1.**
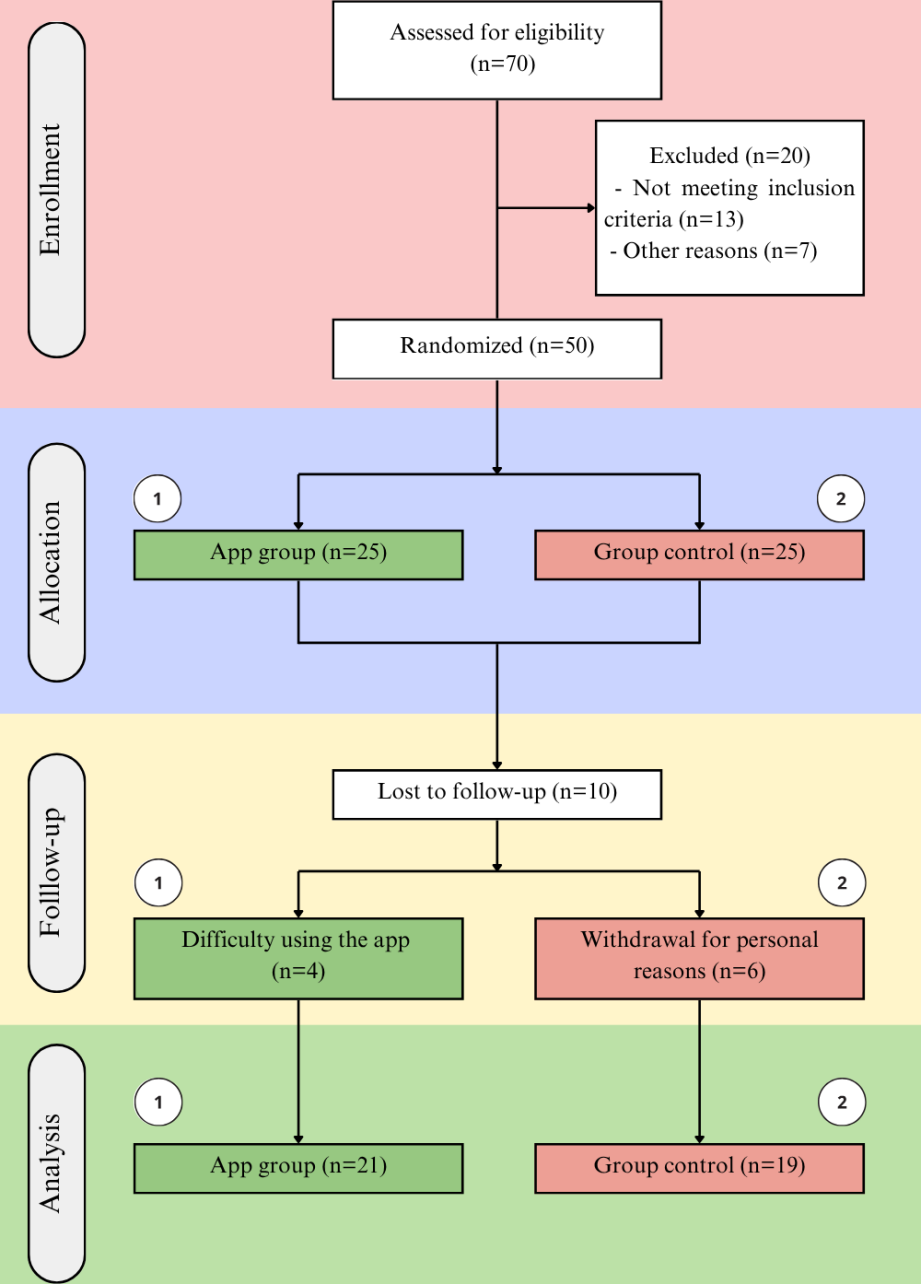
CONSORT (Consolidated Standards of Reporting Trials) 2010 flow diagram. Change in internet navigation skills (MCDMSênior domain score) from baseline to 14-week follow-up among intervention and control groups in a randomized controlled trial of a lifestyle mobile health app for older women; Ribeirão Preto, São Paulo, Brazil. Error bars represent the standard error of the mean.

This study involved a sample of 40 older adult women, aged between 59 and 69 years. The app group included 21 participants, while the control group was composed of 19 individuals (Figure 1). The sociodemographic variables of both groups presented a balanced distribution.

No significant difference in mean age was found between the groups (app: mean 63.95, SD 3.15 y; control: mean 64.32, SD 2.64 y; *t*_38_=−0.392; *P*=.70). Similarly, no significant difference was observed in years of schooling (app: mean 12.14, SD 3.11 y; control: mean 12.58, SD 2.87 y; *t*_38_=−0.559; *P*=.58). Despite differences in mean socioeconomic scores, both groups were classified in category A, the highest bracket in the Brazilian Economic Classification Criteria, with A2 for the app group and A1 for the control group.

Digital competencies were assessed using a semistructured questionnaire based on the MCDMSênior, administered before and after the intervention in the app and control groups. Table 1 presents the detailed results, including mean and SD for each competence at pre- and postintervention, Cohen *d* effect sizes, *F* test, and *P* values from the GLMM analyses, and model fit indices (AIC).

**Table 1.** Baseline sociodemographic and digital literacy characteristics of older women (≥60  y) enrolled in a 14-week randomized controlled trial evaluating a lifestyle mobile health app; Ribeirão Preto, São Paulo, Brazil. Group differences tested using independent *t* tests or chi-square tests, as appropriate. Comparison of the effects of the app on digital skills and cognition between the app and control groups. *P*<.05 represents the statistical significance of the observed differences.

Variables	App group (n=21), mean (SD)			Control group (n=19), mean (SD)			Time		Group		Time × group		Wald chi-square (*df*)	*P* value	Standardized β coefficient (CI)	AIC^a^
	Pre	Post	Cohen *d*	Pre	Post	Cohen *d*	*F* test (*df*)	*P* value	*F* test (*df*)	*P* value	*F* test (*df*)	*P* value				
Competence 1^b^	3.90 (1.30)	3.66 (0.85)	0.219	3.52 (0.77)	4.00 (1.05)	0.521	0.347 (1, 38)	.56	0.008 (1, 38)	.93	3.172 (1, 38)	.08	1.42 (38)	.15	0.245 (0.06-0.97)	234.0
Competence 2^c^	5.28 (1.90)	4.23 (1.37)	0.634	4.26 (1.44)	4.15 (0.95)	0.090	7.329 (1, 38)	.01	1.774 (1, 38)	.19	4.897 (1, 38)	.03	3.24 (38)	2.95	1.257 (0.68-2.30)	269.3
Competence 3^d^	5.95 (2.01)	5.71 (2.28)	0.112	4.84 (1.86)	4.47 (1.34)	0.228	0.980 (1, 38)	.33	5.002 (1, 38)	.03	0.045 (1, 38)	.83	2.72 (38)	.35	1.819 (0.88-3.73)	322.2
Competence 4^e^	4.76 (1.64)	4.61 (1.82)	0.087	4.73 (1.85)	5.52 (1.83)	0.429	1.018 (1, 38)	.32	0.892 (1, 38)	.35	2.116 (1, 38)	.15	2.08 (38)	2.09	1.150 (0.45-2.94)	316.6
Competence 5^f^	4.71 (1.34)	4.42 (1.07)	0.239	4.57 (1.67)	5.05 (1.35)	0.316	0.118 (1, 38)	.73	0.523 (1, 38)	.47	1.934 (1, 38)	.17	1.29 (38)	.20	0.394 (0.08-1.79)	278.0
Competence 6^g^	6.23 (1.70)	6.00 (1.48)	0.144	5.84 (2.00)	5.47 (1.92)	0.189	0.791 (1, 38)	.38	1.054 (1, 38)	.31	0.037 (1, 38)	.85	1.60 (38)	.11	0.853 (0.25-2.89)	318.2

a AIC: Akaike Information Criterion.

b Competence 1: basic knowledge of technology.

c Competence 2: internet navigation skills.

d Competence 3: use of mobile applications.

e Competence 4: ability to carry out online research.

f Competence 5: digital communication.

g Competence 6: use of digital resources for older people.

Significant effects were found for competence 2 (internet navigation skills), with a main effect of time (*F*_1,38_=7.329; *P*=.01) and a significant time×group interaction (*F*_1,38_=4.897; *P*=.03). Competence 3 (use of mobile apps) showed a significant main effect of group (*F*_1,38_=5.002; *P*=.03). These findings indicate that the intervention group improved internet navigation skills, while the control group showed greater gains in basic technology skills (competence 1). The full results are summarized in Table 1.

Significant differences were observed between the intervention and control groups after the intervention period. Competence 2 (internet navigation skills) showed a main effect of time (*F*_1,38_=7.329; *P*=.01) and a significant time×group interaction (*F*_1,38_=4.897; *P*=.03). Competence 3 (use of mobile apps) showed a significant main effect of group (*F*_1,38_=5.002; *P*=.03). Regarding effect sizes, the intervention group demonstrated a moderate effect for competence 2 (Cohen *d*=0.634) and small effects for competences 1 and 5 (Cohen *d*=0.219 and 0.239, respectively). The control group showed a moderate effect for competence 1 (Cohen *d*=0.521) and small effects for competences 4 and 5 (Cohen *d*=0.429 and 0.316, respectively).

Satisfaction with the educational materials, assessed using the SAM and its CVI, was low, with an average SAM score of 35% and CVI of 0.66, classifying the materials as “inadequate or unacceptable.” Usability, measured using the SUS and the CVI, yielded an average SUS score of 71.5 points and a CVI of 0.70, indicating that the usability of the app was rated as “average.”

### Qualitative Results

In addition to the quantitative analyses, qualitative data were collected from participants in the intervention group. These narratives enriched the understanding of the user experience, offering insights into the emotional, cognitive, and social dimensions that influenced the adoption of the mobile app. A thematic content analysis identified 4 central categories:

- Insecurity and fear of judgment: Participants frequently described feelings of insecurity when engaging with the app, particularly when comparing themselves to younger individuals. They expressed frustration about their perceived slowness, fear of mistakes, and embarrassment in asking for help.

*The technology seems complicated. Although the support is great and I appreciate that they thought of me, I worry about appearing incapable in front of younger people. They are so fast with these things, while I take longer, which makes me feel a bit embarrassed.*

[Participant, 63 y old]

- Internalization of ageism and negative self-image: Some participants revealed internalized stereotypes of aging, perceiving themselves as “too old” to master digital skills. This negative self-image reduced motivation to persist, despite recognizing the potential benefits of the app.

*Although the app is accessible, I still struggle with the prejudice I feel because of my age. I feel like these things are not for older people. I am always one step behind, and that discourages me, even though I know I am progressing.*

[Participant, 67 y old].

- Dependence on social support and reluctance to ask for help: Although participants recognized the importance of family and social support, they often felt like a burden, leading to reluctance in asking for assistance. This ambivalence illustrates the tension between valuing support and the desire for autonomy.

*Although the app was highly recommended, I preferred to use it during my walks with new friends so as not to bother my family. My grandchildren are good with technology, but I feel like I am taking their time away from more important activities, especially since they complain and get annoyed.*

[Participant, 64 y old]

- Discrepancy between perceived ease and actual autonomous use: Finally, some participants reported that the app seemed simple when used with guidance, but difficult to manage independently. This discrepancy exposed challenges in achieving digital autonomy.

*When I use it alone, it feels very difficult, but when someone shows me, I end up finding it easy.*

[Participant, 65 y old]

Taken together, the qualitative findings demonstrate that although the mobile app was perceived as accessible and its support system was valued, psychosocial factors—such as insecurity, internalized ageism, and dependence on others—remained strong determinants of technology use. These results suggest that interventions aiming to enhance digital inclusion in older adults should not only focus on simplifying technical features but also actively address emotional and social barriers to autonomy and confidence.

## Discussion

### Principal Findings

The main results showed that the use of the app promoted notable improvements, particularly in internet navigation skills and the use of mobile apps. These gains were accompanied by moderate levels of satisfaction and usability, findings that confirm the potential of digital resources to foster behavioral changes, while also reflecting the persistent technological barriers and attitudes among the older adult population [35,36].

The study reveals uniformity in the sociodemographic characteristics of the groups analyzed, demonstrating the robustness of the research. This consistency reflects the effective randomization of participants, which is crucial for validating the results, minimizing potential bias, and strengthening the credibility of the findings. Consistency in these attributes is essential to ensure that any observed effects are attributable to the interventions and not to external factors [26,37].

The findings show that participants were predominantly from economic class A and had, on average, 12 years of schooling. It is observed that older adults with higher incomes have a greater understanding of digital skills and a greater propensity to adopt technologies and perceive them as useful [38,39]. Furthermore, older adults with higher levels of education are more likely to use smartphones and health apps, corroborating the results of this research [39,40]. Therefore, the results of this study offer a comprehensive analysis of the digital skills assessed in the sample of older adults, revealing specific areas for continuous improvement.

In competence 2, which assesses internet navigation skills, the moderate effect size of Cohen *d* in the intervention group suggests that the app contributed to the development of this skill. Studies show a positive association between internet use and cognitive performance among older adults, highlighting the need for personalized technology training for older adults with varied cognitive abilities and experiences [41,42].

In competence 1, focused on the use of basic technologies, a moderate effect of Cohen *d* was observed in both the intervention group and the control group. This indicates that participants in both groups improved fundamental skills, such as turning on and off the cell phone and installing mobile apps. These skills not only facilitate social interaction and access to health and well-being services but also significantly promote autonomy and digital inclusion [2,43].

In competence 5, which assesses digital communication, a small Cohen *d* effect was observed in both groups, suggesting a low impact on this specific aspect of participants’ digital skills. This underscores the complexity of digital communication among older adults and highlights the continued need for tailored interventions to overcome the specific barriers faced by this age group [42].

Regarding competence 4, which measures the ability to conduct online research, a small Cohen *d* effect was observed in the control group, while in the intervention group, there was no significant difference. However, a more detailed analysis of the raw data revealed a reception about the participants’ practices regarding Internet research. Some research suggests that older adults who seek health information online may risk stress, excessive anxiety, and emotional distress [43].

Thus, this skill increases independence in seeking health information, reduces misinformation, and improves the ability to make one’s own decisions [44]. Digital interventions aimed at educating individuals on selecting reliable online health information have shown significant improvements in participants’ ability to assess the credibility of information. This emphasizes the value of this training in combating misinformation during events such as the COVID-19 pandemic [44].

Regarding the satisfaction findings, carried out by the SAM instrument, they indicated that, despite offering meetings to familiarize themselves with the use of the app, the educational materials provided in the app were considered “inadequate or unacceptable” for the participants. This suggests that the content may be too complex, and not well adapted to the needs of the users, or not presented in a clear and accessible way [25].

However, the difficulty in satisfying older adults regarding the use of mobile apps is not limited to the complexity of the content. Recent studies indicate that lack of familiarity with technology, fear, and insecurity when handling electronic devices, as well as visual, motor, and memory difficulties, are significant barriers for this population [45,46].

The SUS questionnaire assessed the usability of a system, while the content validity index measured the quality of the content. Both revealed “average” results [47]. A complementary study highlights that effective usability involves the integration of design and functionality, ensuring that users can not only operate the app, but also absorb and apply the information provided [48].

The qualitative analysis identified four main categories that directly influenced participants’ experiences with the app: (1) insecurity and fear of judgment, (2) internalization of ageism and negative self-image, (3) dependence on social support coupled with reluctance to ask for help, and (4) discrepancy between perceived ease and effective autonomous use. These findings suggest that, although the app was considered accessible, emotional and social barriers remained substantial obstacles to digital appropriation among older adults. Feelings of inadequacy, hesitation to request assistance, and persistent self-doubt reinforced the impact of internalized ageism, consistent with evidence that negative stereotypes of aging undermine confidence and limit technology adoption [49,50]. Even when technical usability is positively evaluated, psychosocial factors—such as fear of mistakes, embarrassment in seeking help, and the perception of being a burden—constrain the full integration of digital tools into daily life [43,47]. Moreover, the discrepancy observed between perceived ease and actual autonomous use highlights the importance of long-term reinforcement, ongoing support, and personalized digital experiences to bridge the gap between usability in principle and usability in practice [48].

Therefore, it is crucial that developers and educators work together to create content that is both informative and easily digestible for older adults. Integrating user feedback into the design and iterative testing phase can be an effective strategy to achieve this goal. This approach is essential to improving the user experience, ensuring that digital products not only meet functional needs but also promote intuitive and enriching interaction [51].

Despite the significant results, this project has some limitations that must be considered. First, the sample size was small. The complexity of the guidance and technological and technical support during the implementation of the intervention justifies the recommendation of reduced samples for this type of study. Larger samples could make the quality of the support offered unfeasible. Even so, this reduced sample may have limited the generalization of some study outcomes.

Another limitation highlighted is the short intervention period used in the study. Short-term interventions may not fully capture the long-term effects of mobile app use on older adults’ digital skills. It is therefore suggested that future studies include longer follow-up periods to better assess the sustainability and lasting impact of digital interventions.

Additionally, it should be noted that owning a compatible mobile device was an inclusion criterion for participation. While necessary for the feasibility of the intervention, this requirement means that all participants already had at least minimal previous exposure to smartphones. Consequently, baseline familiarity with digital devices may have influenced both initial competence levels and the extent of improvements observed. Future research should consider stratifying participants by their previous device use or digital experience to better isolate the effect of the intervention on those with different starting points.

Furthermore, it should be noted that the final sample consisted exclusively of women. Although recruitment was open to both sexes, only female participants completed the study. This gender bias may be reflected in older women’s greater participation in health promotion programs and greater presence in community groups. For future research, we recommend seeking gender balance in the sample to investigate potential sex differences in the adoption of digital health technologies and implementing specific recruitment strategies to target the male group. Unfortunately, the study did not include more digitally vulnerable older adults, such as those without previous access to smartphones, with low digital literacy, or in areas with limited connectivity. In these cases, barriers to adoption may be greater, requiring strategies such as device availability, offline use, and expanded technical support. Finally, an important recommendation is to apply the results in other scenarios and contexts beyond the one studied. Replicating the study in different settings, such as urban and rural communities, at different socioeconomic and cultural levels, can provide additional insights into the effectiveness of the mobile app in different older adult populations.

### Conclusion

This study critically investigated the impact of a mobile app on improving digital skills among older adults, demonstrating that, despite observed gains in basic technology use, such as internet browsing and online research, considerable challenges remain inherent to the use of mobile devices by this population. The findings emphasize the importance of understanding the difficulties users face, highlighting issues such as ageism, fear of interacting with new technologies, difficulty using them, and reluctance to ask for help. These factors can directly impact usability and user satisfaction, 2 essential dimensions for the effectiveness and acceptance of digital tools, especially when such technologies are used to support the health and well-being needs of older adults. The research suggests that, to promote more robust and effective digital inclusion, it is crucial that app design and user support be refined. This should address not only technical skills but also the emotional and social aspects that influence the adoption and continued use of technology by this age group.

## Supplementary material

10.2196/76725 Checklist 1 CONSORT-EHEALTH (V 1.6.1)

## Abbreviations

AIC: Akaike Information Criterion
CONSORT: Consolidated Standards of Reporting Trials
CVI: content validation index
GLMM: generalized linear mixed models
HIPAA: Health Insurance Portability and Accountability Act
ITT: intention-to-treat
MCDMSênior: *Modelo de Competências Digitais para M-learning com foco em idosos*
mHealth: mobile health
SAM: suitability assessment of material
SUS: System Usability Scale
