# Supplementary material for: Impact of an mHealth App on Digital Transformation: Randomized Clinical Trial on Strengthening Digital Skills in Older Women
Source: JMIR Form Res. 2026 Jun 30;10:e76725. doi: 10.2196/76725 (PMC13318084; doi:10.2196/76725)
Supplement: Checklist 1 [file formative-v10-e76725-s001.pdf]

CONSORT-EHEALTH (V 1.6.1) - Submission/Publication Form

The CONSORT-EHEALTH checklist is intended for authors of randomized trials evaluating web-based and Internet-based applications/interventions, including mobile interventions, electronic games (incl multiplayer games), social media, certain telehealth applications, and other interactive and/or networked electronic applications. Some of the items (e.g. all subitems under item 5 - description of the intervention) may also be applicable for other study designs.

The goal of the CONSORT EHEALTH checklist and guideline is to be
a) a guide for reporting for authors of RCTs,
b) to form a basis for appraisal of an ehealth trial (in terms of validity)

CONSORT-EHEALTH items/subitems are MANDATORY reporting items for studies published in the Journal of Medical Internet Research and other journals / scientific societies endorsing the checklist.

Items numbered 1., 2., 3., 4a., 4b etc are original CONSORT or CONSORT-NPT (non-pharmacologic treatment) items.
Items with Roman numerals (i., ii, iii, iv etc.) are CONSORT-EHEALTH extensions/clarifications.

As the CONSORT-EHEALTH checklist is still considered in a formative stage, we would ask that you also RATE ON A SCALE OF 1-5 how important/useful you feel each item is FOR THE PURPOSE OF THE CHECKLIST and reporting guideline (optional).

Mandatory reporting items are marked with a red *.
In the textboxes, either copy & paste the relevant sections from your manuscript into this form - please include any quotes from your manuscript in QUOTATION MARKS,
or answer directly by providing additional information not in the manuscript, or elaborating on why the item was not relevant for this study.

YOUR ANSWERS WILL BE PUBLISHED AS A SUPPLEMENTARY FILE TO YOUR PUBLICATION IN JMIR AND ARE CONSIDERED PART OF YOUR PUBLICATION (IF ACCEPTED).
Please fill in these questions diligently. Information will not be copyedited, so please use proper spelling and grammar, use correct capitalization, and avoid abbreviations.

DO NOT FORGET TO SAVE AS PDF _AND_ CLICK THE SUBMIT BUTTON SO YOUR ANSWERS ARE IN OUR DATABASE !!!

Citation Suggestion (if you append the pdf as Appendix we suggest to cite this paper in the caption):
Eysenbach G, CONSORT-EHEALTH Group
CONSORT-EHEALTH: Improving and Standardizing Evaluation Reports of Web-based and Mobile Health Interventions
J Med Internet Res 2011;13(4):e126

URL: http://www.jmir.org/2011/4/e126/
doi: 10.2196/jmir.1923
PMID: 22209829

laura.sampaio@estudante.ufscar.br
Mudar de conta

Rascunho não salvo.

Não compartilhado

* Indica uma pergunta obrigatória

Your name *
First Last

Laura Sampaio

Primary Affiliation (short), City, Country *
University of Toronto, Toronto, Canada

Federal University of São Carlos, São Carlos

Your e-mail address *
abc@gmail.com

laura.sampaio@estudante.ufscar.br

Title of your manuscript *
Provide the (draft) title of your manuscript.

Impact of a mobile health application on digital transformation: a randomized clinical trial on strengthening digital skills in older women

Name of your App/Software/Intervention *

If there is a short and a long/alternate name, write the short name first and add the long name in brackets.

Viva (Lifestyle Mobile Health Application)

Evaluated Version (if any)

e.g. "V1", "Release 2017-03-01", "Version 2.0.27913"

Sua resposta

Language(s) *

What language is the intervention/app in? If multiple languages are available, separate by comma (e.g. "English, French")

Portuguese

URL of your Intervention Website or App

e.g. a direct link to the mobile app on app in appstore (itunes, Google Play), or URL of the website. If the intervention is a DVD or hardware, you can also link to an Amazon page.

Sua resposta

URL of an image/screenshot (optional)

Sua resposta

Accessibility *

Can an enduser access the intervention presently?

- ○ access is free and open
- ○ access only for special usergroups, not open
- ○ access is open to everyone, but requires payment/subscription/in-app purchases
- ◉ app/intervention no longer accessible
- ○ Outro:

Primary Medical Indication/Disease/Condition *

e.g. "Stress", "Diabetes", or define the target group in brackets after the condition, e.g. "Autism (Parents of children with)", "Alzheimers (Informal Caregivers of)"

Older women (≥60 years)

Primary Outcomes measured in trial *

comma-separated list of primary outcomes reported in the trial

Improvement in digital competencies (basic te

Secondary/other outcomes

Are there any other outcomes the intervention is expected to affect?

Satisfaction with educational content (Suitability Assessment of Materials score), and System usability (System Usability Scale score).

Recommended "Dose" *

What do the instructions for users say on how often the app should be used?

- (●) Approximately Daily
- ( ) Approximately Weekly
- ( ) Approximately Monthly
- ( ) Approximately Yearly
- ( ) "as needed"
- ( ) Outro:

Approx. Percentage of Users (starters) still using the app as recommended after 3 months *

- (●) unknown / not evaluated
- ( ) 0-10%
- ( ) 11-20%
- ( ) 21-30%
- ( ) 31-40%
- ( ) 41-50%
- ( ) 51-60%
- ( ) 61-70%
- ( ) 71%-80%
- ( ) 81-90%
- ( ) 91-100%
- ( ) Outro:

Overall, was the app/intervention effective? *

- ( ) yes: all primary outcomes were significantly better in intervention group vs control
- (•) partly: SOME primary outcomes were significantly better in intervention group vs control
- ( ) no statistically significant difference between control and intervention
- ( ) potentially harmful: control was significantly better than intervention in one or more outcomes
- ( ) inconclusive: more research is needed
- ( ) Outro:

Article Preparation Status/Stage *

At which stage in your article preparation are you currently (at the time you fill in this form)

- ( ) not submitted yet - in early draft status
- ( ) not submitted yet - in late draft status, just before submission
- ( ) submitted to a journal but not reviewed yet
- (•) submitted to a journal and after receiving initial reviewer comments
- ( ) submitted to a journal and accepted, but not published yet
- ( ) published
- ( ) Outro:

Journal *

If you already know where you will submit this paper (or if it is already submitted), please provide the journal name (if it is not JMIR, provide the journal name under "other")

- ○ not submitted yet / unclear where I will submit this
- ○ Journal of Medical Internet Research (JMIR)
- ○ JMIR mHealth and UHealth
- ○ JMIR Serious Games
- ○ JMIR Mental Health
- ○ JMIR Public Health
- ◉ JMIR Formative Research
- ○ Other JMIR sister journal
- ○ Outro:

Is this a full powered effectiveness trial or a pilot/feasibility trial? *

- ○ Pilot/feasibility
- ◉ Fully powered

Manuscript tracking number *

If this is a JMIR submission, please provide the manuscript tracking number under "other" (The ms tracking number can be found in the submission acknowledgement email, or when you login as author in JMIR. If the paper is already published in JMIR, then the ms tracking number is the four-digit number at the end of the DOI, to be found at the bottom of each published article in JMIR)

- ○ no ms number (yet) / not (yet) submitted to / published in JMIR
- ◉ Outro: 76725

TITLE AND ABSTRACT

1a) TITLE: Identification as a randomized trial in the title

1a) Does your paper address CONSORT item 1a? *

I.e does the title contain the phrase "Randomized Controlled Trial"? (if not, explain the reason under "other")

- (x) yes
- ( ) Outro:

1a-i) Identify the mode of delivery in the title

Identify the mode of delivery. Preferably use “web-based” and/or “mobile” and/or “electronic game” in the title. Avoid ambiguous terms like “online”, “virtual”, “interactive”. Use “Internet-based” only if Intervention includes non-web-based Internet components (e.g. email), use “computer-based” or “electronic” only if offline products are used. Use “virtual” only in the context of “virtual reality” (3-D worlds). Use “online” only in the context of “online support groups”. Complement or substitute product names with broader terms for the class of products (such as “mobile” or “smart phone” instead of “iphone”), especially if the application runs on different platforms.

	1	2	3	4	5	
subitem not at all important	( )	( )	( )	( )	(x)	essential

Limpar seleção

Does your paper address subitem 1a-i? *

Copy and paste relevant sections from manuscript title (include quotes in quotation marks "like this" to indicate direct quotes from your manuscript), or elaborate on this item by providing additional information not in the ms, or briefly explain why the item is not applicable/relevant for your study

Impact of a “mobile health application” on “digital transformation”: a randomized clinical trial on strengthening “digital skills” in older women

1a-ii) Non-web-based components or important co-interventions in title

Mention non-web-based components or important co-interventions in title, if any (e.g., "with telephone support").

	1	2	3	4	5	
subitem not at all important	○	○	○	○	◉	essential

Limpar seleção

Does your paper address subitem 1a-ii?

Copy and paste relevant sections from manuscript title (include quotes in quotation marks "like this" to indicate direct quotes from your manuscript), or elaborate on this item by providing additional information not in the ms, or briefly explain why the item is not applicable/relevant for your study

"Impact" of a mobile health application on digital transformation: a "randomized clinical trial" on "strengthening" digital skills in older women

1a-iii) Primary condition or target group in the title

Mention primary condition or target group in the title, if any (e.g., "for children with Type I Diabetes") Example: A Web-based and Mobile Intervention with Telephone Support for Children with Type I Diabetes: Randomized Controlled Trial

	1	2	3	4	5	
subitem not at all important	○	○	○	○	◉	essential

Limpar seleção

Does your paper address subitem 1a-iii? *

Copy and paste relevant sections from manuscript title (include quotes in quotation marks "like this" to indicate direct quotes from your manuscript), or elaborate on this item by providing additional information not in the ms, or briefly explain why the item is not applicable/relevant for your study

Impact of a mobile health application on digital transformation: a randomized clinical trial on strengthening digital skills in “older women”

1b) ABSTRACT: Structured summary of trial design, methods, results, and conclusions

NPT extension: Description of experimental treatment, comparator, care providers, centers, and blinding status.

1b-i) Key features/functionalities/components of the intervention and comparator in the METHODS section of the ABSTRACT

Mention key features/functionalities/components of the intervention and comparator in the abstract. If possible, also mention theories and principles used for designing the site. Keep in mind the needs of systematic reviewers and indexers by including important synonyms. (Note: Only report in the abstract what the main paper is reporting. If this information is missing from the main body of text, consider adding it)

	1	2	3	4	5	
subitem not at all important	○	○	○	○	◉	essential

Limpar seleção

Does your paper address subitem 1b-i? *

Copy and paste relevant sections from the manuscript abstract (include quotes in quotation marks "like this" to indicate direct quotes from your manuscript), or elaborate on this item by providing additional information not in the ms, or briefly explain why the item is not applicable/relevant for your study

“Objective: To evaluate the impact of a lifestyle mobile health application on improving digital skills, as well as to analyze the level of satisfaction and usability of the application.”

1b-ii) Level of human involvement in the METHODS section of the ABSTRACT

Clarify the level of human involvement in the abstract, e.g., use phrases like “fully automated” vs. “therapist/nurse/care provider/physician-assisted” (mention number and expertise of providers involved, if any). (Note: Only report in the abstract what the main paper is reporting. If this information is missing from the main body of text, consider adding it)

	1	2	3	4	5	
subitem not at all important	○	○	○	○	◉	essential

Limpar seleção

Does your paper address subitem 1b-ii?

Copy and paste relevant sections from the manuscript abstract (include quotes in quotation marks "like this" to indicate direct quotes from your manuscript), or elaborate on this item by providing additional information not in the ms, or briefly explain why the item is not applicable/relevant for your study

“Methods: A 14-week randomized clinical trial was conducted in Ribeirão Preto, São Paulo, Brazil. Forty elderly women were randomized into an intervention group (n=21), who used the mobile application, and a control group (n=19).”

1b-iii) Open vs. closed, web-based (self-assessment) vs. face-to-face assessments in the METHODS section of the ABSTRACT

Mention how participants were recruited (online vs. offline), e.g., from an open access website or from a clinic or a closed online user group (closed usergroup trial), and clarify if this was a purely web-based trial, or there were face-to-face components (as part of the intervention or for assessment). Clearly say if outcomes were self-assessed through questionnaires (as common in web-based trials). Note: In traditional offline trials, an open trial (open-label trial) is a type of clinical trial in which both the researchers and participants know which treatment is being administered. To avoid confusion, use "blinded" or "unblinded" to indicated the level of blinding instead of "open", as "open" in web-based trials usually refers to "open access" (i.e. participants can self-enrol). (Note: Only report in the abstract what the main paper is reporting. If this information is missing from the main body of text, consider adding it)

	1	2	3	4	5	
subitem not at all important	○	○	○	○	◉	essential

Limpar seleção

Does your paper address subitem 1b-iii?

Copy and paste relevant sections from the manuscript abstract (include quotes in quotation marks "like this" to indicate direct quotes from your manuscript), or elaborate on this item by providing additional information not in the ms, or briefly explain why the item is not applicable/relevant for your study

"Methods: A 14-week randomized clinical trial was conducted in Ribeirão Preto, São Paulo, Brazil. Forty elderly women were randomized into an intervention group (n=21), who used the mobile application, and a control group (n=19). Digital competencies were measured before and after the intervention using a semi-structured questionnaire based on the MCDMSenior framework, covering six domains: basic technology use, internet navigation, mobile application use, online research, digital communication, and utilization of digital resources. Additionally, satisfaction with the educational content was evaluated using the Suitability Assessment of Materials (SAM) and system usability was assessed using the System Usability Scale (SUS). Qualitative data were collected through semi-structured, in-person interviews conducted immediately after the intervention with all intervention participants."

1b-iv) RESULTS section in abstract must contain use data

Report number of participants enrolled/assessed in each group, the use/uptake of the intervention (e.g., attrition/adherence metrics, use over time, number of logins etc.), in addition to primary/secondary outcomes. (Note: Only report in the abstract what the main paper is reporting. If this information is missing from the main body of text, consider adding it)

	1	2	3	4	5	
subitem not at all important	○	○	○	○	◉	essential

Limpar seleção

Does your paper address subitem 1b-iv?

Copy and paste relevant sections from the manuscript abstract (include quotes in quotation marks "like this" to indicate direct quotes from your manuscript), or elaborate on this item by providing additional information not in the ms, or briefly explain why the item is not applicable/relevant for your study

"The application group included 21 participants, while the control group was composed of 19 individuals. Post-intervention analyses revealed significant differences in specific digital competencies. The intervention group demonstrated a moderate improvement in internet navigation skills, while gains in basic technology use and digital communication were minimal. Conversely, the control group exhibited moderate improvement in basic technology skills and lower effects in online research and digital communication. Overall, satisfaction with the educational content was low, and usability was rated as average."

1b-v) CONCLUSIONS/DISCUSSION in abstract for negative trials

Conclusions/Discussions in abstract for negative trials: Discuss the primary outcome - if the trial is negative (primary outcome not changed), and the intervention was not used, discuss whether negative results are attributable to lack of uptake and discuss reasons. (Note: Only report in the abstract what the main paper is reporting. If this information is missing from the main body of text, consider adding it)

	1	2	3	4	5	
subitem not at all important	○	○	○	○	◉	essential

Limpar seleção

Does your paper address subitem 1b-v?

Copy and paste relevant sections from the manuscript abstract (include quotes in quotation marks "like this" to indicate direct quotes from your manuscript), or elaborate on this item by providing additional information not in the ms, or briefly explain why the item is not applicable/relevant for your study

"Mobile health applications can effectively enhance certain digital competencies in older women, particularly internet navigation, but improvements in content suitability and usability are needed. Refinements in design and tailored support are essential to overcome age-related barriers and foster digital inclusion."

INTRODUCTION

2a) In INTRODUCTION: Scientific background and explanation of rationale

2a-i) Problem and the type of system/solution

Describe the problem and the type of system/solution that is object of the study: intended as stand-alone intervention vs. incorporated in broader health care program? Intended for a particular patient population? Goals of the intervention, e.g., being more cost-effective to other interventions, replace or complement other solutions? (Note: Details about the intervention are provided in "Methods" under 5)

	1	2	3	4	5	
subitem not at all important	○	○	○	○	◉	essential

Limpar seleção

Does your paper address subitem 2a-i? *

Copy and paste relevant sections from the manuscript (include quotes in quotation marks "like this" to indicate direct quotes from your manuscript), or elaborate on this item by providing additional information not in the ms, or briefly explain why the item is not applicable/relevant for your study

"Despite these advances, the elderly population still faces significant barriers in adopting digital technologies. Studies highlight previous experience, digital exclusion, cognitive and motivational barriers. Other critical obstacles are usability and user satisfaction, which affect digital adoption among seniors. Therefore, it is essential to address these barriers with targeted interventions and support to overcome the digital divide and facilitate older adults' engagement with digital skills. Digital competencies are defined as skills, knowledge, and attitudes crucial to the effective use of digital technologies, from basic device operation to evaluating and communicating online content, benefiting the health and well-being of the elderly population. Recent evidence demonstrates that higher levels of digital competence are consistently associated with increased adoption of telehealth services across different contexts and populations. Such competence not only facilitates access to online health resources but also enhances users' confidence in navigating digital systems, thereby reducing the risk of exclusion and mistrust. Moreover, systematic reviews highlight that insufficient digital skills remain a primary barrier for older adults, reinforcing that targeted strategies to strengthen digital literacy are crucial to mitigate inequities in access and health outcomes. Thus, this research specifically investigates how a mobile application that promotes lifestyle habits can improve digital skills in the elderly, in addition to analyzing usability and user satisfaction."

2a-ii) Scientific background, rationale: What is known about the (type of) system

Scientific background, rationale: What is known about the (type of) system that is the object of the study (be sure to discuss the use of similar systems for other conditions/diagnoses, if appropiate), motivation for the study, i.e. what are the reasons for and what is the context for this specific study, from which stakeholder viewpoint is the study performed, potential impact of findings [2]. Briefly justify the choice of the comparator.

	1	2	3	4	5	
subitem not at all important	○	○	○	○	◉	essential

Limpar seleção

Does your paper address subitem 2a-ii? *

Copy and paste relevant sections from the manuscript (include quotes in quotation marks "like this" to indicate direct quotes from your manuscript), or elaborate on this item by providing additional information not in the ms, or briefly explain why the item is not applicable/relevant for your study

"Mobile health (mHealth) applications are a potential strategy to promote digital inclusion, strengthen digital competencies, and support healthy aging. Nonetheless, studies show that culturally adapted, multidisciplinary interventions for this group remain scarce and are rarely assessed through both quantitative and qualitative methods."
"Digital competencies are defined as skills, knowledge, and attitudes crucial to the effective use of digital technologies, from basic device operation to evaluating and communicating online content, benefiting the health and well-being of the elderly population. Recent evidence demonstrates that higher levels of digital competence are consistently associated with increased adoption of telehealth services across different contexts and populations. Such competence not only facilitates access to online health resources but also enhances users' confidence in navigating digital systems, thereby reducing the risk of exclusion and mistrust. Moreover, systematic reviews highlight that insufficient digital skills remain a primary barrier for older adults, reinforcing that targeted strategies to strengthen digital literacy are crucial to mitigate inequities in access and health outcomes."
"This research specifically investigates how a mobile application that promotes lifestyle habits can improve digital skills in the elderly, in addition to analyzing usability and user satisfaction."

2b) In INTRODUCTION: Specific objectives or hypotheses

Does your paper address CONSORT subitem 2b? *

Copy and paste relevant sections from the manuscript (include quotes in quotation marks "like this" to indicate direct quotes from your manuscript), or elaborate on this item by providing additional information not in the ms, or briefly explain why the item is not applicable/relevant for your study

"Thus, this research specifically investigates how a mobile application that promotes lifestyle habits can improve digital skills in the elderly, in addition to analyzing usability and user satisfaction."

METHODS

3a) Description of trial design (such as parallel, factorial) including allocation ratio

Does your paper address CONSORT subitem 3a? *

Copy and paste relevant sections from the manuscript (include quotes in quotation marks "like this" to indicate direct quotes from your manuscript), or elaborate on this item by providing additional information not in the ms, or briefly explain why the item is not applicable/relevant for your study

"This project is a fragment of a larger research project, the objective of which is to develop and validate a multidisciplinary application to promote changes in lifestyle habits, with simple guidelines, according to the daily demands of each elderly person. The research is a randomized clinical trial, with a 14-week intervention. Participants were randomly assigned to two groups: the application group and the control group."
"Participants were randomly allocated to the intervention and control groups in a 1:1 ratio using a computer-generated randomization sequence (Microsoft Excel RAND function). Randomization was performed independently by a researcher who was not involved in recruitment or outcome assessment to ensure allocation concealment. The sequence was generated prior to the start of the study, and group assignments were revealed only after baseline assessments were completed. No stratification was used, given the homogeneity of the sample in terms of age range and socioeconomic characteristics."

3b) Important changes to methods after trial commencement (such as eligibility criteria), with reasons

Does your paper address CONSORT subitem 3b? *

Copy and paste relevant sections from the manuscript (include quotes in quotation marks "like this" to indicate direct quotes from your manuscript), or elaborate on this item by providing additional information not in the ms, or briefly explain why the item is not applicable/relevant for your study

The manuscript does not report any major changes in methods after the start of the clinical trial. Therefore, CONSORT subsection 3b does not apply to this study.

3b-i) Bug fixes, Downtimes, Content Changes

Bug fixes, Downtimes, Content Changes: ehealth systems are often dynamic systems. A description of changes to methods therefore also includes important changes made on the intervention or comparator during the trial (e.g., major bug fixes or changes in the functionality or content) (5-iii) and other “unexpected events” that may have influenced study design such as staff changes, system failures/downtimes, etc. [2].

	1	2	3	4	5	
subitem not at all important	○	○	○	○	◉	essential

Limpar seleção

Does your paper address subitem 3b-i?

Copy and paste relevant sections from the manuscript (include quotes in quotation marks "like this" to indicate direct quotes from your manuscript), or elaborate on this item by providing additional information not in the ms, or briefly explain why the item is not applicable/relevant for your study

The manuscript does not report any bug fixes, downtimes, content changes, or other unexpected events affecting the intervention or comparator during the trial. Therefore, subitem 3b-i is not applicable to this study.

4a) Eligibility criteria for participants

Does your paper address CONSORT subitem 4a? *

Copy and paste relevant sections from the manuscript (include quotes in quotation marks "like this" to indicate direct quotes from your manuscript), or elaborate on this item by providing additional information not in the ms, or briefly explain why the item is not applicable/relevant for your study

The inclusion criteria were "The elderly individuals aged 60 years or older who owned a mobile device compatible with the application. The application was developed for both Android and iOS operating systems, and it was tested on all models owned by participants, functioning without technical restrictions. Internet connectivity (via Wi-Fi or mobile data) was required to access the educational content and interactive features of the application, ensuring full usability and synchronization of data. The exclusion criteria included limitations in carrying out the assessments included in the project, such as visual and auditory limitations and severe cognitive impairments, assessed by the Montreal Cognitive Assessment (MoCA) instrument."

4a-i) Computer / Internet literacy

Computer / Internet literacy is often an implicit “de facto” eligibility criterion - this should be explicitly clarified.

	1	2	3	4	5	
subitem not at all important	○	○	○	○	◉	essential

Limpar seleção

Does your paper address subitem 4a-i?

Copy and paste relevant sections from the manuscript (include quotes in quotation marks "like this" to indicate direct quotes from your manuscript), or elaborate on this item by providing additional information not in the ms, or briefly explain why the item is not applicable/relevant for your study

Connectivity (via Wi-Fi or mobile data) was required to access the educational content and interactive features of the application, ensuring full usability and synchronization of data."

4a-ii) Open vs. closed, web-based vs. face-to-face assessments:

Open vs. closed, web-based vs. face-to-face assessments: Mention how participants were recruited (online vs. offline), e.g., from an open access website or from a clinic, and clarify if this was a purely web-based trial, or there were face-to-face components (as part of the intervention or for assessment), i.e., to what degree got the study team to know the participant. In online-only trials, clarify if participants were quasi-anonymous and whether having multiple identities was possible or whether technical or logistical measures (e.g., cookies, email confirmation, phone calls) were used to detect/prevent these.

	1	2	3	4	5	
subitem not at all important	○	○	○	○	◉	essential

Limpar seleção

Does your paper address subitem 4a-ii? *

Copy and paste relevant sections from the manuscript (include quotes in quotation marks "like this" to indicate direct quotes from your manuscript), or elaborate on this item by providing additional information not in the ms, or briefly explain why the item is not applicable/relevant for your study

"Participants were recruited through posters posted in strategic locations, social media outreach, and press coverage. Interested participants called the research team, expressed their interest, and verified their eligibility."
"This fragment of the study was conducted as a mixed-methods design, integrating quantitative and qualitative approaches..." and included "semi-structured, in-person interviews conducted immediately after the intervention with all intervention participants."
The study was not purely web-based — it combined in-person components (for baseline assessments, app introduction meetings, ongoing support, and post-intervention interviews) with app-based activities. Participants were known to the research team and were not quasi-anonymous.

4a-iii) Information giving during recruitment

Information given during recruitment. Specify how participants were briefed for recruitment and in the informed consent procedures (e.g., publish the informed consent documentation as appendix, see also item X26), as this information may have an effect on user self-selection, user expectation and may also bias results.

	1	2	3	4	5	
subitem not at all important	○	○	○	○	◉	essential

Limpar seleção

Does your paper address subitem 4a-iii?

Copy and paste relevant sections from the manuscript (include quotes in quotation marks "like this" to indicate direct quotes from your manuscript), or elaborate on this item by providing additional information not in the ms, or briefly explain why the item is not applicable/relevant for your study

The participants called the research team, expressed their interest, and verified their eligibility."
"All participants"Interested signed the Informed Consent Form (ICF), being informed about the research, its minimal risks, and their rights, including the option to withdraw without consequences. To ensure the privacy and confidentiality of participants, all data collected was anonymized and disidentified before analysis. Personal information was treated with the utmost confidentiality, and access to the raw data was restricted to authorized research staff only. There was no financial or other compensation for study participants. Participation was voluntary and based on an interest in contributing to the research."

4b) Settings and locations where the data were collected

Does your paper address CONSORT subitem 4b? *

Copy and paste relevant sections from the manuscript (include quotes in quotation marks "like this" to indicate direct quotes from your manuscript), or elaborate on this item by providing additional information not in the ms, or briefly explain why the item is not applicable/relevant for your study

"The research was carried out in elderly people living in the city of Ribeirão Preto, in the interior of the state of São Paulo, Brazil. It was carried out between July and December 2023."
"The qualitative interviews were conducted after the end of the intervention period, while the questionnaires and interviews took place at the University of São Paulo in Ribeirão Preto (USP), the institution where the research originated."

4b-i) Report if outcomes were (self-)assessed through online questionnaires

Clearly report if outcomes were (self-)assessed through online questionnaires (as common in web-based trials) or otherwise.

	1	2	3	4	5	
subitem not at all important	○	○	○	○	●	essential

Limpar seleção

Does your paper address subitem 4b-i? *

Copy and paste relevant sections from the manuscript (include quotes in quotation marks "like this" to indicate direct quotes from your manuscript), or elaborate on this item by providing additional information not in the ms, or briefly explain why the item is not applicable/relevant for your study

The competencies were "Digital measured before and after the intervention using a semi-structured questionnaire based on the MCDMSenior framework, covering six domains: basic technology use, internet navigation, mobile application use, online research, digital communication, and utilization of digital resources."
"All participants in both groups completed the pre- and post-assessment instruments, except for SAM and SUS, as the control group did app. Monitoring not use the intervention and contact with participants were carried out to assist in understanding the application, minimize the risk of bias due to lack of familiarity, and reduce potential dropouts. This justification also applied to the qualitative interviews. The qualitative interviews were conducted after the end of the intervention questionnaires and period, while the interviews took in São Paulo in place at the University Ribeirão Preto (USP), the institution where the research originated."

4b-ii) Report how institutional affiliations are displayed

Report how institutional affiliations are displayed to potential participants [on ehealth media], as affiliations with prestigious hospitals or universities may affect volunteer rates, use, and reactions with regards to an intervention.(Not a required item – describe only if this may bias results)

	1	2	3	4	5	
subitem not at all important	○	○	○	○	●	essential

Limpar seleção

Does your paper address subitem 4b-ii?

Copy and paste relevant sections from the manuscript (include quotes in quotation marks "like this" to indicate direct quotes from your manuscript), or elaborate on this item by providing additional information not in the ms, or briefly explain why the item is not applicable/relevant for your study

The manuscript does not report how institutional affiliations were displayed to potential participants on eHealth media. Therefore, subitem 4b-ii is not applicable to this study.

5) The interventions for each group with sufficient details to allow replication, including how and when they were actually administered

5-i) Mention names, credential, affiliations of the developers, sponsors, and owners

Mention names, credential, affiliations of the developers, sponsors, and owners [6] (if authors/evaluators are owners or developer of the software, this needs to be declared in a "Conflict of interest" section or mentioned elsewhere in the manuscript).

	1	2	3	4	5	
subitem not at all important	○	○	○	○	●	essential

Limpar seleção

Does your paper address subitem 5-i?

Copy and paste relevant sections from the manuscript (include quotes in quotation marks "like this" to indicate direct quotes from your manuscript), or elaborate on this item by providing additional information not in the ms, or briefly explain why the item is not applicable/relevant for your study

The manuscript states:
Funding and sponsorship:
"This project was supported by the National Council for Scientific and Technological Development (CNPq); and by the São Paulo Research Foundation (FAPESP) - No. 2023/16552-4. The funders had no role in the study design, data collection, data analysis, interpretation of results, or writing of the manuscript."
Developers/owners:
"The best details of the application steps can be read in an article developed by Sobrinho and his collaborators (2024)."
Conflict of interest:
"The authors declare that they have no conflict of interest."

5-ii) Describe the history/development process

Describe the history/development process of the application and previous formative evaluations (e.g., focus groups, usability testing), as these will have an impact on adoption/use rates and help with interpreting results.

	1	2	3	4	5	
subitem not at all important	○	○	○	○	●	essential

Limpar seleção

Does your paper address subitem 5-ii?

Copy and paste relevant sections from the manuscript (include quotes in quotation marks "like this" to indicate direct quotes from your manuscript), or elaborate on this item by providing additional information not in the ms, or briefly explain why the item is not applicable/relevant for your study

"This project is a fragment of a larger research project, the objective of which is to develop and validate a multidisciplinary application to promote changes in lifestyle habits, with simple guidelines, according to the daily demands of each elderly person."
"At the beginning of the intervention, a detailed meeting was held to introduce the application, explore its functionalities and clarify operational doubts. During the first week, the researchers maintained daily contact with the intervention group, offering ongoing support to facilitate familiarization with the mobile application. The frequency of contact was adjusted to every other day in the second week and subsequently to biweekly intervals, aiming to reduce technical difficulties and assess independent adherence to the application interface."
"This monitoring strategy was designed to overcome barriers to use due to digital limitations and to monitor genuine participant adherence."
"Preliminary pilot testing also indicated that participants were able to understand and respond to the items appropriately, supporting its feasibility in this context."

5-iii) Revisions and updating

Revisions and updating. Clearly mention the date and/or version number of the application/intervention (and comparator, if applicable) evaluated, or describe whether the intervention underwent major changes during the evaluation process, or whether the development and/or content was “frozen” during the trial. Describe dynamic components such as news feeds or changing content which may have an impact on the replicability of the intervention (for unexpected events see item 3b).

	1	2	3	4	5	
subitem not at all important	○	○	○	○	◉	essential

Limpar seleção

Does your paper address subitem 5-iii?

Copy and paste relevant sections from the manuscript (include quotes in quotation marks "like this" to indicate direct quotes from your manuscript), or elaborate on this item by providing additional information not in the ms, or briefly explain why the item is not applicable/relevant for your study

"The application was developed for both Android and iOS operating systems, and it was tested on all models owned by participants, functioning without technical restrictions. Internet connectivity (via Wi-Fi or mobile data) was required to access the educational content and interactive features of the application, ensuring full usability and synchronization of data."
"At the beginning of the intervention, a detailed meeting was held to introduce the application, explore its functionalities and clarify operational doubts. During the first week, the researchers maintained daily contact with the intervention group, offering ongoing support to facilitate familiarization with the mobile application. The frequency of contact was adjusted to every other day in the second week and subsequently to biweekly intervals, aiming to reduce technical difficulties and assess independent adherence to the application interface."

5-iv) Quality assurance methods

Provide information on quality assurance methods to ensure accuracy and quality of information provided [1], if applicable.

	1	2	3	4	5	
subitem not at all important	○	○	○	○	◉	essential

Limpar seleção

Does your paper address subitem 5-iv?

Copy and paste relevant sections from the manuscript (include quotes in quotation marks "like this" to indicate direct quotes from your manuscript), or elaborate on this item by providing additional information not in the ms, or briefly explain why the item is not applicable/relevant for your study

"Participants' satisfaction with the app's educational content was measured using the Suitability Assessment of Materials (SAM), originally developed in English and later translated and adapted to Portuguese. The SAM was included to evaluate whether the educational content provided in the application was understandable, culturally appropriate, and motivating for older adults, thereby ensuring that usability findings could be interpreted in light of content adequacy."
"To complement this assessment, the Content Validation Index (CVI) was calculated to quantify inter-rater agreement regarding the relevance and clarity of each SAM item. The CVI was introduced to provide an additional layer of rigor, ensuring that the judgments about the educational content were not only based on participant feedback but also on expert consensus. This content analysis was performed both with a focus group and with participants from the intervention group, ensuring a comprehensive and comparative assessment of item validity."
"The CVI classifies scores into six levels: unacceptable (≤ 0.50), poor (> 0.50 and ≤ 0.60), mediocre (> 0.60 and ≤ 0.70), average (>0.70 and ≤0.80), good (>0.80 and ≤0.90), and excellent (> 0.90). This approach allowed the identification of areas for improvement in the educational material and ensured robust interpretation of the data obtained."
"User experience with the application was assessed using the System Usability Scale (SUS), originally developed by John Brooke and adapted for the Brazilian context… In parallel, the CVI was also used to assess the consistency of the SUS items, applying the same content validity classification as described for the SAM."

5-v) Ensure replicability by publishing the source code, and/or providing screenshots/screen-capture video, and/or providing flowcharts of the algorithms used

Ensure replicability by publishing the source code, and/or providing screenshots/screen-capture video, and/or providing flowcharts of the algorithms used. Replicability (i.e., other researchers should in principle be able to replicate the study) is a hallmark of scientific reporting.

	1	2	3	4	5	
subitem not at all important	○	○	○	○	◉	essential

Limpar seleção

Does your paper address subitem 5-v?

Copy and paste relevant sections from the manuscript (include quotes in quotation marks "like this" to indicate direct quotes from your manuscript), or elaborate on this item by providing additional information not in the ms, or briefly explain why the item is not applicable/relevant for your study

"The data that support the findings of this study are not publicly available due to ethical and privacy restrictions. However, the datasets are available from the corresponding author upon reasonable request via email. The code used for data analysis is available from the corresponding author upon request."

5-vi) Digital preservation

Digital preservation: Provide the URL of the application, but as the intervention is likely to change or disappear over the course of the years; also make sure the intervention is archived (Internet Archive, webcitation.org, and/or publishing the source code or screenshots/videos alongside the article). As pages behind login screens cannot be archived, consider creating demo pages which are accessible without login.

	1	2	3	4	5	
subitem not at all important	○	○	○	○	●	essential

Limpar seleção

Does your paper address subitem 5-vi?

Copy and paste relevant sections from the manuscript (include quotes in quotation marks "like this" to indicate direct quotes from your manuscript), or elaborate on this item by providing additional information not in the ms, or briefly explain why the item is not applicable/relevant for your study

"The data that support the findings of this study are not publicly available due to ethical and privacy restrictions. However, the datasets are available from the corresponding author upon reasonable request via email. The code used for data analysis is available from the corresponding author upon request."

5-vii) Access

Access: Describe how participants accessed the application, in what setting/context, if they had to pay (or were paid) or not, whether they had to be a member of specific group. If known, describe how participants obtained “access to the platform and Internet” [1]. To ensure access for editors/reviewers/readers, consider to provide a “backdoor” login account or demo mode for reviewers/readers to explore the application (also important for archiving purposes, see vi).

	1	2	3	4	5	
subitem not at all important	○	○	○	○	◉	essential

Limpar seleção

Does your paper address subitem 5-vii? *

Copy and paste relevant sections from the manuscript (include quotes in quotation marks "like this" to indicate direct quotes from your manuscript), or elaborate on this item by providing additional information not in the ms, or briefly explain why the item is not applicable/relevant for your study

"The research was carried out in elderly people living in the city of Ribeirão Preto, in the interior of the state of São Paulo, Brazil. It was carried out between July and December 2023. Participants were recruited through posters posted in strategic locations, social media outreach, and press coverage. Interested participants called the research team, expressed their interest, and verified their eligibility."
"The inclusion criteria were elderly individuals aged 60 years or older who owned a mobile device compatible with the application. The application was developed for both Android and iOS operating systems, and it was tested on all models owned by participants, functioning without technical restrictions. Internet connectivity (via Wi-Fi or mobile data) was required to access the educational content and interactive features of the application, ensuring full usability and synchronization of data."
"There was no financial or other compensation for study participants. Participation was voluntary and based on an interest in contributing to the research."
The manuscript does not mention any requirement to be a member of a specific group beyond the inclusion criteria, nor does it provide a “backdoor” login account, demo mode, or similar access for editors/reviewers/readers.

5-viii) Mode of delivery, features/functionalities/components of the intervention and comparator, and the theoretical framework

Describe mode of delivery, features/functionalities/components of the intervention and comparator, and the theoretical framework [6] used to design them (instructional strategy [1], behaviour change techniques, persuasive features, etc., see e.g., [7, 8] for terminology). This includes an in-depth description of the content (including where it is coming from and who developed it) [1]," whether [and how] it is tailored to individual circumstances and allows users to track their progress and receive feedback" [6]. This also includes a description of communication delivery channels and – if computer-mediated communication is a component – whether communication was synchronous or asynchronous [6]. It also includes information on presentation strategies [1], including page design principles, average amount of text on pages, presence of hyperlinks to other resources, etc. [1].

	1	2	3	4	5	
subitem not at all important	○	○	○	○	◉	essential

Limpar seleção

Does your paper address subitem 5-viii? *

Copy and paste relevant sections from the manuscript (include quotes in quotation marks "like this" to indicate direct quotes from your manuscript), or elaborate on this item by providing additional information not in the ms, or briefly explain why the item is not applicable/relevant for your study

"This project is a fragment of a larger research project, the objective of which is to develop and validate a multidisciplinary application to promote changes in lifestyle habits, with simple guidelines, according to the daily demands of each elderly person."
"The application was developed for both Android and iOS operating systems, and it was tested on all models owned by participants, functioning without technical restrictions. Internet connectivity (via Wi-Fi or mobile data) was required to access the educational content and interactive features of the application, ensuring full usability and synchronization of data."
"At the beginning of the intervention, a detailed meeting was held to introduce the application, explore its functionalities and clarify operational doubts. During the first week, the researchers maintained daily contact with the intervention group, offering ongoing support to facilitate familiarization with the mobile application. The frequency of contact was adjusted to every other day in the second week and subsequently to biweekly intervals, aiming to reduce technical difficulties and assess independent adherence to the application interface."
"This monitoring strategy was designed to overcome barriers to use due to digital limitations and to monitor genuine participant adherence."
"A semi-structured questionnaire based on the MCDMSenior framework was applied to assess the impact on improving the digital skills of the participants… It encompasses six domains, each of which was analyzed separately in this study: (1) Basic knowledge of technology… (2) Internet navigation skills… (3) Use of mobile applications… (4) Ability to conduct online research… (5) Digital communication… and (6) Use of digital resources… The choice of this model is justified by its conceptual breadth and its direct alignment with the digital skills mobilized during the use of the application, such as navigation, interaction, and consumption of educational content."
"Participants' satisfaction with the app's educational content was measured using the Suitability Assessment of Materials (SAM)… The SAM consists of a checklist with 30 items, divided into six categories: content, text comprehension, illustration, presentation, motivation, and cultural adaptation."
"User experience with the application was assessed using the System Usability Scale (SUS)… Composed of 10 items, the SUS investigates aspects such as ease of use, speed of learning, efficiency, and overall satisfaction with the system."

5-ix) Describe use parameters

Describe use parameters (e.g., intended “doses” and optimal timing for use). Clarify what instructions or recommendations were given to the user, e.g., regarding timing, frequency, heaviness of use, if any, or was the intervention used ad libitum.

	1	2	3	4	5	
subitem not at all important	○	○	○	○	◉	essential

Limpar seleção

Does your paper address subitem 5-ix?

Copy and paste relevant sections from the manuscript (include quotes in quotation marks "like this" to indicate direct quotes from your manuscript), or elaborate on this item by providing additional information not in the ms, or briefly explain why the item is not applicable/relevant for your study

Sua resposta

5-x) Clarify the level of human involvement

Clarify the level of human involvement (care providers or health professionals, also technical assistance) in the e-intervention or as co-intervention (detail number and expertise of professionals involved, if any, as well as “type of assistance offered, the timing and frequency of the support, how it is initiated, and the medium by which the assistance is delivered”. It may be necessary to distinguish between the level of human involvement required for the trial, and the level of human involvement required for a routine application outside of a RCT setting (discuss under item 21 – generalizability).

	1	2	3	4	5	
subitem not at all important	○	○	○	○	◉	essential

Limpar seleção

Does your paper address subitem 5-x?

Copy and paste relevant sections from the manuscript (include quotes in quotation marks "like this" to indicate direct quotes from your manuscript), or elaborate on this item by providing additional information not in the ms, or briefly explain why the item is not applicable/relevant for your study

Sua resposta

5-xi) Report any prompts/reminders used

Report any prompts/reminders used: Clarify if there were prompts (letters, emails, phone calls, SMS) to use the application, what triggered them, frequency etc. It may be necessary to distinguish between the level of prompts/reminders required for the trial, and the level of prompts/reminders for a routine application outside of a RCT setting (discuss under item 21 – generalizability).

	1	2	3	4	5	
subitem not at all important	○	○	○	○	◉	essential

Limpar seleção

Does your paper address subitem 5-xi? *

Copy and paste relevant sections from the manuscript (include quotes in quotation marks "like this" to indicate direct quotes from your manuscript), or elaborate on this item by providing additional information not in the ms, or briefly explain why the item is not applicable/relevant for your study

"At the beginning of the intervention, a detailed meeting was held to introduce the application, explore its functionalities and clarify operational doubts. During the first week, the researchers maintained daily contact with the intervention group, offering ongoing support to facilitate familiarization with the mobile application. The frequency of contact was adjusted to every other day in the second week and subsequently to biweekly intervals, aiming to reduce technical difficulties and assess independent adherence to the application interface."
"This monitoring strategy was designed to overcome barriers to use due to digital limitations and to monitor genuine participant adherence."

5-xii) Describe any co-interventions (incl. training/support)

Describe any co-interventions (incl. training/support): Clearly state any interventions that are provided in addition to the targeted eHealth intervention, as ehealth intervention may not be designed as stand-alone intervention. This includes training sessions and support [1]. It may be necessary to distinguish between the level of training required for the trial, and the level of training for a routine application outside of a RCT setting (discuss under item 21 – generalizability.

	1	2	3	4	5	
subitem not at all important	○	○	○	○	◉	essential

Limpar seleção

Does your paper address subitem 5-xii? *

Copy and paste relevant sections from the manuscript (include quotes in quotation marks "like this" to indicate direct quotes from your manuscript), or elaborate on this item by providing additional information not in the ms, or briefly explain why the item is not applicable/relevant for your study

"At the beginning of the intervention, a detailed meeting was held to introduce the application, explore its functionalities and clarify operational doubts. During the first week, the researchers maintained daily contact with the intervention group, offering ongoing support to facilitate familiarization with the mobile application. The frequency of contact was adjusted to every other day in the second week and subsequently to biweekly intervals, aiming to reduce technical difficulties and assess independent adherence to the application interface."
"This monitoring strategy was designed to overcome barriers to use due to digital limitations and to monitor genuine participant adherence."
"The app was free, with doctors and nutritionists available. I took advantage of what I could use on my own, which was not much for someone like me." (Participant, 61 years old)

6a) Completely defined pre-specified primary and secondary outcome measures, including how and when they were assessed

Does your paper address CONSORT subitem 6a? *

Copy and paste relevant sections from the manuscript (include quotes in quotation marks "like this" to indicate direct quotes from your manuscript), or elaborate on this item by providing additional information not in the ms, or briefly explain why the item is not applicable/relevant for your study

"Digital competencies were measured before and after the intervention using a semi-structured questionnaire based on the MCDMSenior framework, covering six domains: basic technology use, internet navigation, mobile application use, online research, digital communication, and utilization of digital resources."
"Participants' satisfaction with the app's educational content was measured using the Suitability Assessment of Materials (SAM), originally developed in English and later translated and adapted to Portuguese."
"User experience with the application was assessed using the System Usability Scale (SUS), originally developed by John Brooke and adapted for the Brazilian context."
"All participants in both groups completed the pre- and post-assessment instruments, except for SAM and SUS, as the control group did not use the intervention app."
These sections define the primary outcome measures (digital competencies across six domains) and secondary outcome measures (satisfaction with educational content via SAM, usability via SUS), and specify how (validated/adapted instruments) and when (before and after the 14-week intervention) they were assessed.

6a-i) Online questionnaires: describe if they were validated for online use and apply CHERRIES items to describe how the questionnaires were designed/deployed

If outcomes were obtained through online questionnaires, describe if they were validated for online use and apply CHERRIES items to describe how the questionnaires were designed/deployed [9].

	1	2	3	4	5	
subitem not at all important	○	○	○	○	◉	essential

Limpar seleção

Does your paper address subitem 6a-i?

Copy and paste relevant sections from manuscript text

"A semi-structured questionnaire based on the MCDMSenior framework was applied to assess the impact on improving the digital skills of the participants. This model was originally developed to evaluate digital competencies in older adults in mobile learning and e-health contexts, with specific emphasis on inclusion and age-related challenges [14]... The choice of this model is justified by its conceptual breadth and its direct alignment with the digital skills mobilized during the use of the application, such as navigation, interaction, and consumption of educational content."
"Although the MCDMSênior was not yet a formally validated instrument at the time of data collection, its theoretical adherence and practical relevance provided strong justification for its use in this study. Preliminary pilot testing also indicated that participants were able to understand and respond to the items appropriately, supporting its feasibility in this context."

6a-ii) Describe whether and how “use” (including intensity of use/dosage) was defined/measured/monitored

Describe whether and how “use” (including intensity of use/dosage) was defined/measured/monitored (logins, logfile analysis, etc.). Use/adoption metrics are important process outcomes that should be reported in any ehealth trial.

	1	2	3	4	5	
subitem not at all important	○	○	○	○	◉	essential

Limpar seleção

Does your paper address subitem 6a-ii?

Copy and paste relevant sections from manuscript text

Sua resposta

6a-iii) Describe whether, how, and when qualitative feedback from participants was obtained

Describe whether, how, and when qualitative feedback from participants was obtained (e.g., through emails, feedback forms, interviews, focus groups).

	1	2	3	4	5	
subitem not at all important	○	○	○	○	◉	essential

Limpar seleção

Does your paper address subitem 6a-iii?

Copy and paste relevant sections from manuscript text

"Qualitative data were collected through semi-structured, in-person interviews conducted immediately after the intervention with all intervention participants. Interviews explored perceptions of the app's usability, satisfaction with its content, barriers, and facilitators to engagement, and perceived changes in digital skills. All interviews were audio-recorded, transcribed, and analyzed thematically by two independent researchers using an inductive coding approach."
"The qualitative interviews were conducted after the end of the intervention period, while the questionnaires and interviews took place at the University of São Paulo in Ribeirão Preto (USP), the institution where the research originated."
These sections describe that qualitative feedback was obtained in person, immediately after the intervention, via semi-structured interviews with all intervention participants, and analyzed thematically.

6b) Any changes to trial outcomes after the trial commenced, with reasons

Does your paper address CONSORT subitem 6b? *

Copy and paste relevant sections from the manuscript (include quotes in quotation marks "like this" to indicate direct quotes from your manuscript), or elaborate on this item by providing additional information not in the ms, or briefly explain why the item is not applicable/relevant for your study

This study did not undergo any changes to the trial outcomes after the trial commenced.

7a) How sample size was determined

NPT: When applicable, details of whether and how the clustering by care provides or centers was addressed

7a-i) Describe whether and how expected attrition was taken into account when calculating the sample size

Describe whether and how expected attrition was taken into account when calculating the sample size.

	1	2	3	4	5	
subitem not at all important	○	○	○	○	◉	essential

Limpar seleção

Does your paper address subitem 7a-i?

Copy and paste relevant sections from manuscript title (include quotes in quotation marks "like this" to indicate direct quotes from your manuscript), or elaborate on this item by providing additional information not in the ms, or briefly explain why the item is not applicable/relevant for your study

"The sample size of causes and effects with a minimum sample size of 34 people was calculated using the G*Power 3.1 software, using ANOVA for inter-intragroup interaction with the following input parameters: effect size of 0.25; type I error equal to 0.05; type II error equal to 0.80; and number of groups equal to 2 and number of measurements equal to 2. A standard correlation between the measurements of 0.50 and a dropout rate of 20% were also used to account for possible sample losses."

7b) When applicable, explanation of any interim analyses and stopping guidelines

Does your paper address CONSORT subitem 7b? *

Copy and paste relevant sections from the manuscript (include quotes in quotation marks "like this" to indicate direct quotes from your manuscript), or elaborate on this item by providing additional information not in the ms, or briefly explain why the item is not applicable/relevant for your study

No interim analyses were conducted, and no stopping guidelines were established for this trial.

8a) Method used to generate the random allocation sequence
NPT: When applicable, how care providers were allocated to each trial group

Does your paper address CONSORT subitem 8a? *

Copy and paste relevant sections from the manuscript (include quotes in quotation marks "like this" to indicate direct quotes from your manuscript), or elaborate on this item by providing additional information not in the ms, or briefly explain why the item is not applicable/relevant for your study

Participants: Answer were randomly allocated to the intervention and control groups in a 1:1 ratio using a computer-generated randomization sequence RAND function). (Microsoft Excel Randomization was by a researcher performed independently who was not involved in recruitment or outcome assessment to ensure allocation concealment. The sequence was generated prior to the start of the study, and group assignments were revealed only after baseline assessments were completed. No stratification was used, given the homogeneity of the sample in terms of age range and socioeconomic characteristics."

8b) Type of randomisation; details of any restriction (such as blocking and block size)

Does your paper address CONSORT subitem 8b? *

Copy and paste relevant sections from the manuscript (include quotes in quotation marks "like this" to indicate direct quotes from your manuscript), or elaborate on this item by providing additional information not in the ms, or briefly explain why the item is not applicable/relevant for your study

Participants were randomly allocated to the intervention and control groups in a 1:1 ratio using a computer-generated randomization sequence (Microsoft Excel RAND function). Randomization was performed independently by a researcher who was not involved in recruitment or outcome assessment to ensure allocation concealment. The sequence was generated prior to the start of the study, and group assignments were revealed only after baseline assessments were completed. No stratification was used, given the homogeneity of the sample in terms of age range and socioeconomic characteristics.
The paper specifies the type of randomization — simple randomization using a computer-generated sequence — and explicitly notes that no restrictions (such as blocking or stratification) were applied. Therefore, CONSORT subitem 8b is addressed, and there were no block sizes or other constraints to report.

9) Mechanism used to implement the random allocation sequence (such as sequentially numbered containers), describing any steps taken to conceal the sequence until interventions were assigned

Does your paper address CONSORT subitem 9? *

Copy and paste relevant sections from the manuscript (include quotes in quotation marks "like this" to indicate direct quotes from your manuscript), or elaborate on this item by providing additional information not in the ms, or briefly explain why the item is not applicable/relevant for your study

"Participants were randomly allocated to the intervention and control groups in a 1:1 ratio using a computer-generated randomization sequence (Microsoft Excel RAND function). Randomization was performed independently by a researcher who was not involved in recruitment or outcome assessment to ensure allocation concealment. The sequence was generated prior to the start of the study, and group assignments were revealed only after baseline assessments were completed."
The paper describes that allocation concealment was maintained by having an independent researcher—separate from recruitment and assessment—generate and hold the randomization sequence. Group assignments were baseline data collection, ensuring that neither participants nor disclosed only after foresee allocation before enrollment assessors could was complete.

10) Who generated the random allocation sequence, who enrolled participants, and who assigned participants to interventions

Does your paper address CONSORT subitem 10? *

Copy and paste relevant sections from the manuscript (include quotes in quotation marks "like this" to indicate direct quotes from your manuscript), or elaborate on this item by providing additional information not in the ms, or briefly explain why the item is not applicable/relevant for your study

"Participants were randomly allocated to the intervention and control groups. Answer in a 1:1 ratio using a computer-generated (Microsoft Excel randomization sequence Randomization was RAND function). Performed independently by a researcher who was not involved in recruitment or outcome assessment to ensure allocation concealment. The prior to the start of the study, and sequence was generated group assignments were revealed only after baseline assessments were completed."
The text specifies who generated the random allocation sequence — an independent researcher not involved in recruitment or outcome assessment.
It also identifies who enrolled participants — the research team that recruited eligible participants through posters, social media, and press coverage, and verified eligibility via phone contact.
Who assigned participants to interventions researcher, using the pre-generated concealed sequence, assigned participants to either the intervention or control group after baseline assessments.

11a) If done, who was blinded after assignment to interventions (for example, participants, care providers, those assessing outcomes) and how

NPT: Whether or not administering co-interventions were blinded to group assignment

11a-i) Specify who was blinded, and who wasn’t

Specify who was blinded, and who wasn’t. Usually, in web-based trials it is not possible to blind the participants [1, 3] (this should be clearly acknowledged), but it may be possible to blind outcome assessors, those doing data analysis or those administering co-interventions (if any).

	1	2	3	4	5	
subitem not at all important	○	○	○	○	◉	essential

Limpar seleção

Does your paper address subitem 11a-i? *

Copy and paste relevant sections from the manuscript (include quotes in quotation marks "like this" to indicate direct quotes from your manuscript), or elaborate on this item by providing additional information not in the ms, or briefly explain why the item is not applicable/relevant for your study

The manuscript does not explicitly state who was blinded after assignment to interventions. There is no direct mention of blinding of participants, care providers, outcome assessors, or data analysts of the intervention.
Given the nature — a mobile health application used by the intervention group — blinding of participants, as they would, was not feasible be aware of whether they were using the app.
"Participants to the intervention and control groups in a 1:1 ratio using a computer-generated were randomly allocated randomization sequence... Randomization was performed independently by a researcher who was not involved in recruitment or outcome assessment to ensure allocation concealment."

11a-ii) Discuss e.g., whether participants knew which intervention was the "intervention of interest" and which one was the "comparator"

Informed consent procedures (4a-ii) can create biases and certain expectations - discuss e.g., whether participants knew which intervention was the "intervention of interest" and which one was the "comparator".

	1	2	3	4	5	
subitem not at all important	○	○	○	○	◉	essential

Limpar seleção

Does your paper address subitem 11a-ii?

Copy and paste relevant sections from the manuscript (include quotes in quotation marks "like this" to indicate direct quotes from your manuscript), or elaborate on this item by providing additional information not in the ms, or briefly explain why the item is not applicable/relevant for your study

Sua resposta

11b) If relevant, description of the similarity of interventions
(this item is usually not relevant for ehealth trials as it refers to similarity of a placebo or sham intervention to a active medication/intervention)

Does your paper address CONSORT subitem 11b? *

Copy and paste relevant sections from the manuscript (include quotes in quotation marks "like this" to indicate direct quotes from your manuscript), or elaborate on this item by providing additional information not in the ms, or briefly explain why the item is not applicable/relevant for your study

This item is not applicable to the present study. The trial compared an active mobile health application intervention with a control group that did not receive the application, rather than comparing two interventions designed to appear similar (e.g., active vs. placebo/sham).

12a) Statistical methods used to compare groups for primary and secondary outcomes
NPT: When applicable, details of whether and how the clustering by care providers or centers was addressed

Does your paper address CONSORT subitem 12a? *

Copy and paste relevant sections from the manuscript (include quotes in quotation marks "like this" to indicate direct quotes from your manuscript), or elaborate on this item by providing additional information not in the ms, or briefly explain why the item is not applicable/relevant for your study

Data were processed and analyzed using IBM SPSS Statistics 25.0 and Microsoft Excel 2019. For a comprehensive view of the data, measures of central tendency and dispersion, such as mean and standard deviation (SD), were calculated, as well as percentages. Initial similarity between groups was analyzed using Student's t-test for socioeconomic variables, years of education, and age.
An intention-to-treat (ITT) analysis was performed using multiple imputations of up to five levels to address missing data and maintain the ITT principle. In this approach, all participants were included according to their original randomization, regardless of treatment adherence or loss to follow-up. Multiple imputation was applied to create five complete data sets, thus minimizing potential bias due to missing data.
To assess time (pre vs. post effects), group (intervention vs. control), and the interaction between them (time x group) on outcome measures, generalized linear mixed models (GLMMs) were used. GLMMs were chosen because they are suitable for analyzing repeated measures data, account for intra-individual correlations, and are robust to imbalanced data sets and missing values.
Adjustment for potential confounders was performed to increase model precision and control for variables known to influence both digital competence and technology adoption among older adults. Specifically, age, sex, education, and socioeconomic status were included as covariates. Model fit was assessed using the Akaike Information Criterion (AIC). All analyses were conducted with 95% confidence intervals and a 5% significance level. For multiple comparisons, Bonferroni post-hoc tests were applied for interpretation. To inform clinical Cohen's d, effect sizes were calculated and categorized as small (d = 0.2), medium (d = 0.5), large (d = 0.8), or very large (d > 1.0).

12a-i) Imputation techniques to deal with attrition / missing values

Imputation techniques to deal with attrition / missing values: Not all participants will use the intervention/comparator as intended and attrition is typically high in ehealth trials. Specify how participants who did not use the application or dropped out from the trial were treated in the statistical analysis (a complete case analysis is strongly discouraged, and simple imputation techniques such as LOCF may also be problematic [4]).

	1	2	3	4	5	
subitem not at all important	○	○	○	○	◉	essential

Limpar seleção

Does your paper address subitem 12a-i? *

Copy and paste relevant sections from the manuscript (include quotes in quotation marks "like this" to indicate direct quotes from your manuscript), or elaborate on this item by providing additional information not in the ms, or briefly explain why the item is not applicable/relevant for your study

"An intention-to-treat (ITT) analysis was performed using up to five-level multiple imputations data and preserve to address missing the ITT principle, all participants. In this approach were included according to their original of treatment adherence or losses to follow randomization, regardless-up. Multiple imputation was applied to create five complete datasets, thereby minimizing potential bias due to missing data. This procedure was adopted because ITT analyses provide a more conservative and realistic estimate of intervention effects in clinical trials."
This section confirms that multiple imputation — not complete case analysis or simple imputation like LOCF — was used to handle attrition and missing values, ensuring all randomized participants were included in the analysis.

12b) Methods for additional analyses, such as subgroup analyses and adjusted analyses

Does your paper address CONSORT subitem 12b? *

Copy and paste relevant sections from the manuscript (include quotes in quotation marks "like this" to indicate direct quotes from your manuscript), or elaborate on this item by providing additional information not in the ms, or briefly explain why the item is not applicable/relevant for your study

Yes. The manuscript describes the methods for additional analyses, including adjusted analyses.
“Adjustment for potential confounders was conducted to increase model precision and control for variables known to influence both digital competence and technology adoption among older adults. Specifically, age, sex, education, and socioeconomic status were included as covariates, given their established associations with digital literacy and health technology use.” No subgroup analyses are reported beyond these adjusted analyses.

X26) REB/IRB Approval and Ethical Considerations [recommended as subheading under "Methods"] (not a CONSORT item)

X26-i) Comment on ethics committee approval

	1	2	3	4	5	
subitem not at all important	○	○	○	○	◉	essential

Limpar seleção

Does your paper address subitem X26-i?

Copy and paste relevant sections from the manuscript (include quotes in quotation marks "like this" to indicate direct quotes from your manuscript), or elaborate on this item by providing additional information not in the ms, or briefly explain why the item is not applicable/relevant for your study

"The study followed the principles of the Declaration of Helsinki. The research was ethically approved by the Research Ethics Committee of the University of São Paulo (USP) under registration number CAAE 58433922.0.0000.5659 and registered with the Brazilian Clinical Trials Registry (ReBEC) under number RBR-6wgkzs8. All participants signed the Informed Consent Form (ICF), being informed about the research, its minimal risks, and their rights, including the option to withdraw without consequences."

x26-ii) Outline informed consent procedures

Outline informed consent procedures e.g., if consent was obtained offline or online (how? Checkbox, etc.?), and what information was provided (see 4a-ii). See [6] for some items to be included in informed consent documents.

	1	2	3	4	5	
subitem not at all important	○	○	○	○	◉	essential

Limpar seleção

Does your paper address subitem X26-ii?

Copy and paste relevant sections from the manuscript (include quotes in quotation marks "like this" to indicate direct quotes from your manuscript), or elaborate on this item by providing additional information not in the ms, or briefly explain why the item is not applicable/relevant for your study

Sua resposta

X26-iii) Safety and security procedures

Safety and security procedures, incl. privacy considerations, and any steps taken to reduce the likelihood or detection of harm (e.g., education and training, availability of a hotline)

	1	2	3	4	5	
subitem not at all important	○	○	○	○	◉	essential

Limpar seleção

Does your paper address subitem X26-iii?

Copy and paste relevant sections from the manuscript (include quotes in quotation marks "like this" to indicate direct quotes from your manuscript), or elaborate on this item by providing additional information not in the ms, or briefly explain why the item is not applicable/relevant for your study

"To ensure the privacy and confidentiality of participants, all data collected was anonymized and disidentified before analysis. Personal information was treated with the utmost confidentiality, and access to the raw data was restricted to authorized research staff only. At the beginning of the intervention, a detailed meeting was held to introduce the application, explore its functionalities and clarify operational doubts. During the first week, the researchers maintained daily contact with the intervention group, offering ongoing support to facilitate familiarization with the mobile application. The frequency of contact was adjusted to every other day in the second week and subsequently to biweekly intervals, aiming to reduce technical difficulties and assess independent adherence to the application interface."

These measures ensured participants were trained in safe use of the application, had ongoing access to researcher support, and that their personal data remained secure and confidential.

RESULTS

13a) For each group, the numbers of participants who were randomly assigned, received intended treatment, and were analysed for the primary outcome
NPT: The number of care providers or centers performing the intervention in each group and the number of patients treated by each care provider in each center

Does your paper address CONSORT subitem 13a? *

Copy and paste relevant sections from the manuscript (include quotes in quotation marks "like this" to indicate direct quotes from your manuscript), or elaborate on this item by providing additional information not in the ms, or briefly explain why the item is not applicable/relevant for your study

Initially, 70 volunteers were assessed for eligibility; 20 were due to medical records that did not meet the criteria or for other reasons. The 50 eligible participants were randomized into two groups (intervention: n = 25; control: n = 25). There were 10 losses to follow-up in the intervention group, resulting in 40 cases analyzed. The intervention group included 21 participants, while the control group consisted of 19 individuals. The study does not involve multiple providers or centers; all participants were recruited and evaluated in a single research setting; therefore, the NPT extension item regarding the number of providers/centers and patients per provider does not apply.

13b) For each group, losses and exclusions after randomisation, together with reasons

Does your paper address CONSORT subitem 13b? (NOTE: Preferably, this is shown in a CONSORT flow diagram) *

Copy and paste relevant sections from the manuscript (include quotes in quotation marks "like this" to indicate direct quotes from your manuscript), or elaborate on this item by providing additional information not in the ms, or briefly explain why the item is not applicable/relevant for your study

Yes. The manuscript reports the losses and exclusions after randomisation, along with reasons, and presents them in a CONSORT flow diagram.

13b-i) Attrition diagram

Strongly recommended: An attrition diagram (e.g., proportion of participants still logging in or using the intervention/comparator in each group plotted over time, similar to a survival curve) or other figures or tables demonstrating usage/dose/engagement.

	1	2	3	4	5	
subitem not at all important	○	○	○	○	◉	essential

Limpar seleção

Does your paper address subitem 13b-i?

Copy and paste relevant sections from the manuscript or cite the figure number if applicable (include quotes in quotation marks "like this" to indicate direct quotes from your manuscript), or elaborate on this item by providing additional information not in the ms, or briefly explain why the item is not applicable/relevant for your study

Sua resposta

14a) Dates defining the periods of recruitment and follow-up

Does your paper address CONSORT subitem 14a? *

Copy and paste relevant sections from the manuscript (include quotes in quotation marks "like this" to indicate direct quotes from your manuscript), or elaborate on this item by providing additional information not in the ms, or briefly explain why the item is not applicable/relevant for your study

Yes. The manuscript specifies the dates for the recruitment and follow-up periods.
"The research was carried out in elderly people living in the city of Ribeirão Preto, in the interior of the state of São Paulo, Brazil. It was carried out between July and December 2023. Participants were recruited through posters posted in strategic locations, social media outreach, and press coverage."

14a-i) Indicate if critical “secular events” fell into the study period

Indicate if critical “secular events” fell into the study period, e.g., significant changes in Internet resources available or “changes in computer hardware or Internet delivery resources”

	1	2	3	4	5	
subitem not at all important	○	○	○	○	◉	essential

Limpar seleção

Does your paper address subitem 14a-i?

Copy and paste relevant sections from the manuscript (include quotes in quotation marks "like this" to indicate direct quotes from your manuscript), or elaborate on this item by providing additional information not in the ms, or briefly explain why the item is not applicable/relevant for your study

Sua resposta

14b) Why the trial ended or was stopped (early)

Does your paper address CONSORT subitem 14b? *

Copy and paste relevant sections from the manuscript (include quotes in quotation marks "like this" to indicate direct quotes from your manuscript), or elaborate on this item by providing additional information not in the ms, or briefly explain why the item is not applicable/relevant for your study

The manuscript does not report that the trial was stopped early. The study appears to have been conducted and completed as planned over the 14-week intervention period. There is no indication of early termination for efficacy, safety, or other reasons. Since no early stopping occurred, this item is not applicable to the present study.

15) A table showing baseline demographic and clinical characteristics for each group

NPT: When applicable, a description of care providers (case volume, qualification, expertise, etc.) and centers (volume) in each group

Does your paper address CONSORT subitem 15? *

Copy and paste relevant sections from the manuscript (include quotes in quotation marks "like this" to indicate direct quotes from your manuscript), or elaborate on this item by providing additional information not in the ms, or briefly explain why the item is not applicable/relevant for your study

Yes. The manuscript includes a table presenting baseline demographic characteristics for each group.

15-i) Report demographics associated with digital divide issues

In ehealth trials it is particularly important to report demographics associated with digital divide issues, such as age, education, gender, social-economic status, computer/Internet/ehealth literacy of the participants, if known.

	1	2	3	4	5	
subitem not at all important	○	○	○	○	◉	essential

Limpar seleção

Does your paper address subitem 15-i? *

Copy and paste relevant sections from the manuscript (include quotes in quotation marks "like this" to indicate direct quotes from your manuscript), or elaborate on this item by providing additional information not in the ms, or briefly explain why the item is not applicable/relevant for your study

Yes. The manuscript reports several demographics directly related to digital divide issues, including age, education, gender, socioeconomic status, and baseline digital literacy.

16) For each group, number of participants (denominator) included in each analysis and whether the analysis was by original assigned groups

16-i) Report multiple “denominators” and provide definitions

Report multiple “denominators” and provide definitions: Report N’s (and effect sizes) “across a range of study participation [and use] thresholds” [1], e.g., N exposed, N consented, N used more than x times, N used more than y weeks, N participants “used” the intervention/comparator at specific pre-defined time points of interest (in absolute and relative numbers per group). Always clearly define “use” of the intervention.

	1	2	3	4	5	
subitem not at all important	○	○	○	○	◉	essential

Limpar seleção

Does your paper address subitem 16-i? *

Copy and paste relevant sections from the manuscript (include quotes in quotation marks "like this" to indicate direct quotes from your manuscript), or elaborate on this item by providing additional information not in the ms, or briefly explain why the item is not applicable/relevant for your study

The manuscript does not explicitly report multiple denominators across different thresholds of participation or use (e.g., number of participants who used the app more than a certain number of times or weeks). It does, however, provide the main denominators for recruitment, allocation, and analysis, as well as reasons for loss to follow-up, and describes the monitoring strategy to support and assess adherence.

16-ii) Primary analysis should be intent-to-treat

Primary analysis should be intent-to-treat, secondary analyses could include comparing only “users”, with the appropriate caveats that this is no longer a randomized sample (see 18-i).

	1	2	3	4	5	
subitem not at all important	○	○	○	○	◉	essential

Limpar seleção

Does your paper address subitem 16-ii?

Copy and paste relevant sections from the manuscript (include quotes in quotation marks "like this" to indicate direct quotes from your manuscript), or elaborate on this item by providing additional information not in the ms, or briefly explain why the item is not applicable/relevant for your study

Yes. The manuscript explicitly states that the primary analysis followed the intention-to-treat (ITT) principle.

17a) For each primary and secondary outcome, results for each group, and the estimated effect size and its precision (such as 95% confidence interval)

Does your paper address CONSORT subitem 17a? *

Copy and paste relevant sections from the manuscript (include quotes in quotation marks "like this" to indicate direct quotes from your manuscript), or elaborate on this item by providing additional information not in the ms, or briefly explain why the item is not applicable/relevant for your study

Yes. The manuscript reports, for each assessed competence (primary and secondary outcomes), the results for each group, along with effect sizes (Cohen's d) and statistical significance from the GLMM analyses.

17a-i) Presentation of process outcomes such as metrics of use and intensity of use

In addition to primary/secondary (clinical) outcomes, the presentation of process outcomes such as metrics of use and intensity of use (dose, exposure) and their operational definitions is critical. This does not only refer to metrics of attrition (13-b) (often a binary variable), but also to more continuous exposure metrics such as "average session length". These must be accompanied by a technical description how a metric like a "session" is defined (e.g., timeout after idle time) [1] (report under item 6a).

	1	2	3	4	5	
subitem not at all important	○	○	○	○	◉	essential

Limpar seleção

Does your paper address subitem 17a-i?

Copy and paste relevant sections from the manuscript (include quotes in quotation marks "like this" to indicate direct quotes from your manuscript), or elaborate on this item by providing additional information not in the ms, or briefly explain why the item is not applicable/relevant for your study

Sua resposta

17b) For binary outcomes, presentation of both absolute and relative effect sizes is recommended

Does your paper address CONSORT subitem 17b? *

Copy and paste relevant sections from the manuscript (include quotes in quotation marks "like this" to indicate direct quotes from your manuscript), or elaborate on this item by providing additional information not in the ms, or briefly explain why the item is not applicable/relevant for your study

All primary and secondary outcomes in this trial are continuous measures of digital competencies, satisfaction, and usability (e.g., MCDMSenior scores, SAM scores, SUS scores), reported as means ± SD, F-statistics, p-values, and Cohen's d effect sizes. Because no binary outcome variables were collected or analysed, presentation of absolute and relative effect sizes is not applicable to this study.

18) Results of any other analyses performed, including subgroup analyses and adjusted analyses, distinguishing pre-specified from exploratory

Does your paper address CONSORT subitem 18? *

Copy and paste relevant sections from the manuscript (include quotes in quotation marks "like this" to indicate direct quotes from your manuscript), or elaborate on this item by providing additional information not in the ms, or briefly explain why the item is not applicable/relevant for your study

Yes. The manuscript reports adjusted analyses and clearly describes that these were pre-specified. No subgroup analyses are presented, and no exploratory analyses beyond the planned models are reported.

18-i) Subgroup analysis of comparing only users

A subgroup analysis of comparing only users is not uncommon in ehealth trials, but if done, it must be stressed that this is a self-selected sample and no longer an unbiased sample from a randomized trial (see 16-iii).

	1	2	3	4	5	
subitem not at all important	○	○	○	○	◉	essential

Limpar seleção

Does your paper address subitem 18-i?

Copy and paste relevant sections from the manuscript (include quotes in quotation marks "like this" to indicate direct quotes from your manuscript), or elaborate on this item by providing additional information not in the ms, or briefly explain why the item is not applicable/relevant for your study

Sua resposta

19) All important harms or unintended effects in each group

(for specific guidance see CONSORT for harms)

Does your paper address CONSORT subitem 19? *

Copy and paste relevant sections from the manuscript (include quotes in quotation marks "like this" to indicate direct quotes from your manuscript), or elaborate on this item by providing additional information not in the ms, or briefly explain why the item is not applicable/relevant for your study

Yes. The manuscript reports unintended effects and difficulties experienced by participants, although no physical harms or privacy breaches occurred.
"Lost to follow-up (n = 10) — Difficulty using the app (n = 4); Withdrawal for personal reasons (n = 6)."
"Participants frequently described feelings of insecurity when engaging with the app, particularly when comparing themselves to younger individuals. They expressed frustration about their perceived slowness, fear of mistakes, and embarrassment in asking for help."
"Some participants revealed internalized stereotypes of aging, perceiving themselves as 'too old' to master digital skills. This negative self-image reduced motivation to persist, despite recognizing the potential benefits of the app."
"Although the mobile application was perceived as accessible and its support system was valued, psychosocial factors — such as insecurity, internalized ageism, and dependence on others — remained strong determinants of technology use."

19-i) Include privacy breaches, technical problems

Include privacy breaches, technical problems. This does not only include physical "harm" to participants, but also incidents such as perceived or real privacy breaches [1], technical problems, and other unexpected/unintended incidents. "Unintended effects" also includes unintended positive effects [2].

	1	2	3	4	5	
subitem not at all important	○	○	○	○	◉	essential

Limpar seleção

Does your paper address subitem 19-i?

Copy and paste relevant sections from the manuscript (include quotes in quotation marks "like this" to indicate direct quotes from your manuscript), or elaborate on this item by providing additional information not in the ms, or briefly explain why the item is not applicable/relevant for your study

To ensure participant privacy and confidentiality, all collected data was anonymized and disidentified prior to analysis. Personal information was treated with the utmost confidentiality, and access to raw data was restricted to authorized research staff only. Regarding technical issues and unintended effects, the article notes that some participants experienced difficulties using the app, which contributed to dropout: "Loss to follow-up (n = 10); Difficulty using the app (n = 4); Dropping out for personal reasons (n = 6)." The qualitative results also highlight unexpected or unintended effects related to usability and engagement: "Some participants reported that the app seemed straightforward when used with guidance, but difficult to manage independently. This discrepancy exposed challenges in achieving digital autonomy." "Although the mobile app was perceived as accessible and its support system was valued, psychosocial factors—such as insecurity, internalized ageism, and dependence on others—remained strong determinants of technology use."

19-ii) Include qualitative feedback from participants or observations from staff/researchers

Include qualitative feedback from participants or observations from staff/researchers, if available, on strengths and shortcomings of the application, especially if they point to unintended/unexpected effects or uses. This includes (if available) reasons for why people did or did not use the application as intended by the developers.

	1	2	3	4	5	
subitem not at all important	○	○	○	○	◉	essential

Limpar seleção

Does your paper address subitem 19-ii?

Copy and paste relevant sections from the manuscript (include quotes in quotation marks "like this" to indicate direct quotes from your manuscript), or elaborate on this item by providing additional information not in the ms, or briefly explain why the item is not applicable/relevant for your study

Qualitative results indicated that while participants valued clarity of navigation and cultural relevance, persistent age-related fears and insecurities were reported when using digital technologies. Participants highlighted the need for more personalized guidance, ongoing motivational support, and technical adjustments to improve usability and engagement.
(a) Insecurity and fear of judgment — Participants frequently described feelings of insecurity when interacting with the app, especially when comparing themselves to younger individuals. They expressed frustration with perceived slowness, fear of making mistakes, and embarrassment when asking for help. (b) Internalization of ageism and negative self-image — Some participants revealed internalized stereotypes of aging, perceiving themselves as "too old" to master digital skills. This negative self-image reduced motivation to persist, despite recognizing the app's potential benefits. (c) Dependence on social support and reluctance to ask for help — Although participants recognized the importance of family and social support, they often felt burdened, leading to reluctance to ask for help.
(d) Discrepancy between perceived ease and actual autonomous use — Some participants reported that the app seemed simple when used with guidance, but difficult to manage independently. This discrepancy exposed the challenges of achieving digital autonomy.
Taken together, the qualitative results demonstrate that, although the mobile app was perceived as accessible and its support system was valued, psychosocial factors — such as insecurity, internalized ageism, and dependence on others — remained strong determinants of technology use. These results suggest that interventions aimed at increasing digital inclusion in older adults should not only focus on simplifying technical features, but also actively address the emotional and social barriers to autonomy and confidence.

DISCUSSION

22) Interpretation consistent with results, balancing benefits and harms, and considering other relevant evidence

NPT: In addition, take into account the choice of the comparator, lack of or partial blinding, and unequal expertise of care providers or centers in each group

22-i) Restate study questions and summarize the answers suggested by the data, starting with primary outcomes and process outcomes (use)

Restate study questions and summarize the answers suggested by the data, starting with primary outcomes and process outcomes (use).

	1	2	3	4	5	
subitem not at all important	○	○	○	○	◉	essential

Limpar seleção

Does your paper address subitem 22-i? *

Copy and paste relevant sections from the manuscript (include quotes in quotation marks "like this" to indicate direct quotes from your manuscript), or elaborate on this item by providing additional information not in the ms, or briefly explain why the item is not applicable/relevant for your study

"The main results showed that the use of the application promoted notable improvements, particularly in internet navigation skills and the use of mobile applications. These gains were accompanied by moderate levels of satisfaction and usability, findings that confirm the potential of digital resources to foster behavioral changes, while also reflecting the persistent technological barriers and attitudes among the elderly population.
Therefore, the results of this study offer a comprehensive analysis of the digital skills assessed in the sample of elderly people, revealing specific areas for continuous improvement. Regarding the satisfaction findings, carried out by the SAM instrument, they indicated that, despite offering meetings to familiarize the use of the application themselves with, the educational materials provided in the application were considered 'inadequate or unacceptable' for the participants. The SUS questionnaire assessed the usability of a system, while the IVC measured the quality of the content. Both revealed 'average' results."

22-ii) Highlight unanswered new questions, suggest future research

Highlight unanswered new questions, suggest future research.

	1	2	3	4	5	
subitem not at all important	○	○	○	○	◉	essential

Limpar seleção

Does your paper address subitem 22-ii?

Copy and paste relevant sections from the manuscript (include quotes in quotation marks "like this" to indicate direct quotes from your manuscript), or elaborate on this item by providing additional information not in the ms, or briefly explain why the item is not applicable/relevant for your study

Sua resposta

20) Trial limitations, addressing sources of potential bias, imprecision, and, if relevant, multiplicity of analyses

20-i) Typical limitations in ehealth trials

Typical limitations in ehealth trials: Participants in ehealth trials are rarely blinded. Ehealth trials often look at a multiplicity of outcomes, increasing risk for a Type I error. Discuss biases due to non-use of the intervention/usability issues, biases through informed consent procedures, unexpected events.

	1	2	3	4	5	
subitem not at all important	○	○	○	○	◉	essential

Limpar seleção

Does your paper address subitem 20-i? *

Copy and paste relevant sections from the manuscript (include quotes in quotation marks "like this" to indicate direct quotes from your manuscript), or elaborate on this item by providing additional information not in the ms, or briefly explain why the item is not applicable/relevant for your study

"Despite the significant results, this project has some limitations that must be considered. First, the sample size was small. The complexity of the guidance and technological and technical support during the implementation of the intervention justifies the recommendation of reduced samples for this type of study. Larger samples could make the quality of the support offered unfeasible. Even so, this reduced sample may have limited the generalization of some study outcomes."
"Another limitation highlighted is the short intervention period used in the study. Short-term interventions may not fully capture the long-term effects of mobile application use on older adults' digital skills. It is therefore suggested that future studies include longer follow-up periods to better assess the sustainability and lasting impact of digital interventions."
"Additionally, it should be noted that owning a compatible mobile device was an inclusion criterion for participation. While necessary for the feasibility of the intervention, this requirement means that all participants already had at least minimal prior exposure to smartphones. Consequently, baseline familiarity with digital devices may have influenced both initial competence levels and the extent of improvements observed."
"Furthermore, it should be noted that the final sample consisted exclusively of women. Although recruitment was open to both sexes, only female participants completed the study. This gender bias may be reflected in older women's greater participation in health promotion programs and greater presence in community groups."
"The qualitative analysis identified four main categories that directly influenced participants' experiences with the application: (a) insecurity and fear of judgment, (b) internalization of ageism and negative self-image, (c) dependence on social support coupled with reluctance to ask for help, and (d) discrepancy between perceived ease and effective autonomous use. These findings suggest that, although the application was considered accessible, emotional and social barriers remained substantial obstacles to digital appropriation among older adults."

21) Generalisability (external validity, applicability) of the trial findings

NPT: External validity of the trial findings according to the intervention, comparators, patients, and care providers or centers involved in the trial

21-i) Generalizability to other populations

Generalizability to other populations: In particular, discuss generalizability to a general Internet population, outside of a RCT setting, and general patient population, including applicability of the study results for other organizations

	1	2	3	4	5	
subitem not at all important	○	○	○	○	◉	essential

Limpar seleção

Does your paper address subitem 21-i?

Copy and paste relevant sections from the manuscript (include quotes in quotation marks "like this" to indicate direct quotes from your manuscript), or elaborate on this item by providing additional information not in the ms, or briefly explain why the item is not applicable/relevant for your study

"Additionally, it should be noted that owning a compatible mobile device was an inclusion criterion for participation. While necessary for the feasibility of the intervention, this requirement means that all participants already had at least minimal prior exposure to smartphones. Consequently, baseline familiarity with digital devices may have influenced both initial competence levels and the extent of improvements observed. Future research should consider stratifying participants by their previous device use or digital experience to better isolate the effect of the intervention on those with different starting points."
"Furthermore, it should be noted that the final sample consisted exclusively of women. Although recruitment was open to both sexes, only female participants completed the study. This gender bias may be reflected in older women's greater participation in health promotion programs and greater presence in community groups. For future research, we recommend seeking gender balance in the sample to investigate potential sex differences in the adoption of digital health technologies, and employing specific recruitment strategies to target the male group."
"Finally, an important recommendation is to apply the results in other scenarios and contexts beyond the one studied. Replicating the study in different settings, such as urban and rural communities, different socioeconomic and cultural levels, can provide additional insights into the effectiveness of the mobile application in different elderly populations."

The manuscript addresses subitem 21-i by noting that the study population was limited to older women with prior smartphone exposure and from a specific socioeconomic context, which may limit generalizability to the broader internet population or general patient populations. It also explicitly recommends replication in varied settings and with more diverse participant profiles to enhance applicability to other organizations and contexts.

21-ii) Discuss if there were elements in the RCT that would be different in a routine application setting

Discuss if there were elements in the RCT that would be different in a routine application setting (e.g., prompts/reminders, more human involvement, training sessions or other co-interventions) and what impact the omission of these elements could have on use, adoption, or outcomes if the intervention is applied outside of a RCT setting.

	1	2	3	4	5	
subitem not at all important	○	○	○	○	◉	essential

Limpar seleção

Does your paper address subitem 21-ii?

Copy and paste relevant sections from the manuscript (include quotes in quotation marks "like this" to indicate direct quotes from your manuscript), or elaborate on this item by providing additional information not in the ms, or briefly explain why the item is not applicable/relevant for your study

"At the beginning of the intervention, a detailed meeting was held to introduce the application, explore its functionalities and clarify operational doubts. During the first week, the researchers maintained daily contact with the intervention group, offering ongoing support to facilitate familiarization with the mobile application. The frequency of contact was adjusted to every other day in the second week and subsequently to biweekly intervals, aiming to reduce technical difficulties and assess independent adherence to the application interface. This monitoring strategy was designed to overcome barriers to use due to digital limitations and to monitor genuine participant adherence."
"The qualitative analysis identified four main categories that directly influenced participants' experiences with the application: (a) insecurity and fear of judgment, (b) internalization of ageism and negative self-image, (c) dependence on social support coupled with reluctance to ask for help, and (d) discrepancy between perceived ease and effective autonomous use. These findings suggest that, although the application was considered accessible, emotional and social barriers remained substantial obstacles to digital appropriation among older adults… Moreover, the discrepancy observed between perceived ease and actual autonomous use highlights the importance of sustained reinforcement, ongoing support, and personalized digital experiences to bridge the gap between usability in principle and usability in practice."

OTHER INFORMATION

23) Registration number and name of trial registry

Does your paper address CONSORT subitem 23? *

Copy and paste relevant sections from the manuscript (include quotes in quotation marks "like this" to indicate direct quotes from your manuscript), or elaborate on this item by providing additional information not in the ms, or briefly explain why the item is not applicable/relevant for your study

"Trial Registration: Brazilian Registry of Clinical Trials (ReBEC), Registration No. RBR-6wgkzs8."
"The research was ethically approved by the Research Ethics Committee of the University of São Paulo (USP) under registration number CAAE 58433922.0.0000.5659 and registered with the Brazilian Clinical Trials Registry (ReBEC) under number RBR-6wgkzs8."

24) Where the full trial protocol can be accessed, if available

Does your paper address CONSORT subitem 24? *

Cite a Multimedia Appendix, other reference, or copy and paste relevant sections from the manuscript (include quotes in quotation marks "like this" to indicate direct quotes from your manuscript), or elaborate on this item by providing additional information not in the ms, or briefly explain why the item is not applicable/relevant for your study

This item is not addressed in the manuscript.

25) Sources of funding and other support (such as supply of drugs), role of funders

Does your paper address CONSORT subitem 25? *

Copy and paste relevant sections from the manuscript (include quotes in quotation marks "like this" to indicate direct quotes from your manuscript), or elaborate on this item by providing additional information not in the ms, or briefly explain why the item is not applicable/relevant for your study

Funding
This project was supported by the National Council for Scientific and Technological Development (CNPq); and by the São Paulo Research Foundation (FAPESP) - No. 2023/16552-4. The funders had no role in the study design, data collection, data analysis, interpretation of results, or writing of the manuscript.

X27) Conflicts of Interest (not a CONSORT item)

X27-i) State the relation of the study team towards the system being evaluated

In addition to the usual declaration of interests (financial or otherwise), also state the relation of the study team towards the system being evaluated, i.e., state if the authors/evaluators are distinct from or identical with the developers/sponsors of the intervention.

	1	2	3	4	5	
subitem not at all important	○	○	○	○	◉	essential

Limpar seleção

Does your paper address subitem X27-i?

Copy and paste relevant sections from the manuscript (include quotes in quotation marks "like this" to indicate direct quotes from your manuscript), or elaborate on this item by providing additional information not in the ms, or briefly explain why the item is not applicable/relevant for your study

The authors declare that they have no conflict of interest.

About the CONSORT EHEALTH checklist

As a result of using this checklist, did you make changes in your manuscript? *

- ○ yes, major changes
- ◉ yes, minor changes
- ○ no

What were the most important changes you made as a result of using this checklist?

We believe that the method was the most changed and improved section. We added more information to improve the text.

How much time did you spend on going through the checklist INCLUDING making changes in your manuscript *

We believe it took two days to fully review the CONSORT and manuscript.

As a result of using this checklist, do you think your manuscript has improved? *

- ◉ yes
- ○ no
- ○ Outro:

Would you like to become involved in the CONSORT EHEALTH group?

This would involve for example becoming involved in participating in a workshop and writing an "Explanation and Elaboration" document

- ( ) yes
- (•) no
- ( ) Outro:

Limpar seleção

Any other comments or questions on CONSORT EHEALTH

Sua resposta

STOP - Save this form as PDF before you click submit

To generate a record that you filled in this form, we recommend to generate a PDF of this page (on a Mac, simply select "print" and then select "print as PDF") before you submit it.

When you submit your (revised) paper to JMIR, please upload the PDF as supplementary file.

Don't worry if some text in the textboxes is cut off, as we still have the complete information in our database. Thank you!

Final step: Click submit !

Click submit so we have your answers in our database!

Enviar

Limpar formulário

Nunca envie senhas pelo Formulários Google.

Este formulário foi criado fora de seu domínio. - Entre em contato com o proprietário do formulário - Termos de Serviço - Política de Privacidade

Este formulário parece suspeito? Denunciar

Google Formulários
